# Annotated type catalogue of the Bothriembryontidae and Odontostomidae (Mollusca, Gastropoda, Orthalicoidea) in the Natural History Museum, London

**DOI:** 10.3897/zookeys.182.2720

**Published:** 2012-04-10

**Authors:** Abraham S.H. Breure, Jonathan D. Ablett

**Affiliations:** 1 Netherlands Centre for Biodiversity Naturalis, P.O. Box 9517, Leiden, the Netherlands; 2 Natural History Museum, Division of Higher Invertebrates, London, SW7 5BD, UK

**Keywords:** Bothriembryontidae, Odontostomidae, types

## Abstract

The type status is described for specimens of 84 taxa classified within the families Bothriembryontidae and Odontostomidae (superfamily Orthalicoidea) and kept in the Natural History Museum, London. Lectotypes are designated for *Bulimus (Liparus) brazieri* Angas, 1871; *Bulimus broderipii* Sowerby I, 1832; *Bulimus fuligineus* Pfeiffer, 1853; *Helix guarani* d’Orbigny, 1835; *Bulimus (Tomigerus) ramagei* E.A. Smith, 1890; *Helix rhodinostoma* d’Orbigny, 1835; *Bulimus (Bulimulus) ridleyi* E.A. Smith, 1890. The type status of the following taxa is changed to lectotype in accordance with Art. 74.6 ICZN: *Placostylus (Euplacostylus) cylindricus* Fulton, 1907; *Bulimus pyrostomus* Pfeiffer, 1860; *Bulimus turneri* Pfeiffer, 1860. The following taxon is synonymised: *Bulimus oblitus* Reeve, 1848 = *Bahiensis neglectus* (Pfeiffer, 1847).

## Introduction and methods

This is the second paper on the types of Orthalicoidea in the Natural History Museum, London. An earlier paper (Breure and Ablett 2011) has presented the context of the collection, the criteria used for the selection of lectotypes, some biohistorical notes, and a list of type specimens belonging to the Amphibulimidae. The aim of this publication is to supplement the earlier paper with data on the type specimens classified with the Bothriembryontidae and Odontostomidae (sensu Breure and Romero in press).

**Figure 1.**
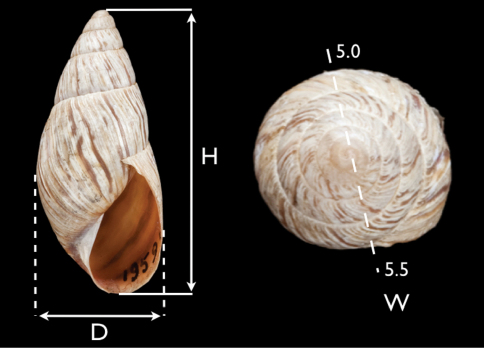
Way the measurements of shell height (H) and diameter (D) have been taken and whorls (W) have been counted.

Abbreviations used for depositories of material are: ANSP, Academy of Natural Sciences, Philadelphia, U.S.A.; MHNG, Muséum d’Histoire Naturelle, Geneve, Switserland; MNHN, Muséum National d’Histoire Naturelle, Paris, France; MNHNM, Museo Nacional de Historia Natural, Montevideo, Uruguay; NHMUK, Natural History Museum, London, U.K.; RBINS, Royal Belgian Institute of Natural Sciences, Brussels, Belgium; RMNH, Netherlands Centre for Biodiversity Naturalis, Leiden, the Netherlands; SMF, Senckenberg Natur-Museum, Frankfurt am Main, Germany; UF, Florida Museum of Natural History, Gainesville, U.S.A.; ZMB, Museum für Naturkunde, Berlin, Germany. Other abbreviations used are: **/** end of line in cited text; coll., collection; D, diameter; H, shell height; leg., *legit*, collected; W, number of whorls. See Fig. 1 for the way measurements of the shell have been taken. Label styles in the Cuming collection (M.C.) are explained in Breure and Ablett (2011: 7–8, fig. 6).

## Systematics

### Systematic list of taxa arranged in generic order

This systematic list follows Breure (1979); the family classification amended as proposed by Breure and Romero (in press). The generic classification has been adapted from Simone (2006) for the Odontostomidae (but see Remarks below); the classification of Bothriembryontidae is based on unpublished data from the senior author. Within each family, genus and at species level, taxa are presented in alphabetical order.

### Family Bothriembryontidae Iredale, 1937

**Remarks.** The Gondwanan group within the Orthalicoidea (Placostylidae sensu Neubert et al. 2009, *Bothriembryon*, *Prestonella*, *Plectostylus*, and *Discoleus*) appears to be monophyletic (Breure and Romero in press). Thus the oldest family name, i.e. Bothriembryontidae, is used herein for this group.

***Aspastus*** Albers, 1850

***Aspastus miltocheilus*** (Reeve, 1848); ***Aspastus sellersi*** (Cox, 1871).

***Bothriembryon*** Pilsbry, 1894

***Bothriembryon angasianus*** (Pfeiffer, 1864); ***Bothriembryon brazieri***(Angas, 1871); ***Bothriembryon dux*** (Pfeiffer, 1861); ***Bothriembryon kingii*** (J.E. Gray, 1825); ***Bothriembryon leeuwinensis*** (E.A. Smith, 1894); ***Bothriembryon physoides*** (Reeve, 1849); ***Bothriembryon rhodostomus*** (J.E. Gray, 1834); ***Bothriembryon spenceri*** (Tate, 1894); ***Bothriembryon tasmanicus*** (Pfeiffer, 1853).

***Callistocharis*** Pilsbry, 1900

***Callistocharis crassilabrum*** (Garrett, 1872); ***Callistocharis ochrostoma*** (Garrett, 1872); *Callistocharis subroseus* Fulton, 1915.

***Eumecostylus*** Martens in Albers, 1860

***Eumecostylus backhuysi*** Delsaerdt, 2010; ***Eumecostylus calus*** (E.A. Smith, 1891); ***Eumecostylus cylindricus*** Fulton, 1907; ***Eumecostylus foxi*** Clench, 1941; ***Eumecostylus hargravesi*** (Cox, 1871); ***Eumecostylus sanchristovalensis*** (Cox, 1870).

***Euplacostylus*** Crosse, 1875

***Euplacostylus koroensis***(Garrett, 1872).

***Placocharis*** Pilsbry, 1900

***Placocharis guppyi*** E.A. Smith, 1891; ***Placocharis kreftii*** (Cox, 1872); ***Placocharis macgillivrayi*** (Pfeiffer, 1855); ***Placocharis strangei*** (Pfeiffer, 1853).

***Placostylus*** Beck, 1837

***Placostylus alba*** (Crosse, 1874); ***Placostylus asopeus*** (Gassies, 1871); ***Placostylus bairdii*** (Reeve, 1848); ***Placostylus bulbulus*** (Gassies, 1871); ***Placostylus corpulentus*** (Gassies, 1871); ***Placostylus cuninculinsulae*** (Cox, 1872); ***Placostylus eddystonensis*** (Pfeiffer, 1855); ***Placostylus edwardsianus*** (Gassies, 1863); ***Placostylus falcicula*** (Gassies, 1871); ***Placostylus gatopensis*** (Crosse, 1870); ***Placostylus grammica*** (Crosse, 1870); ***Placostylus infundibulum*** (Gassies, 1871); ***Placostylus necouensis*** (Gassies, 1871); ***Placostylus ouensis*** (Gassies, 1870); ***Placostylus pinicola*** (Gassies, 1870); ***Placostylus priscus*** Powell, 1938; ***Placostylus pyrostomus*** (Pfeiffer, 1860); ***Placostylus salomonis*** (Pfeiffer, 1853); ***Placostylus savesi*** (Crosse, 1886); ***Placostylus singularis*** (Morelet, 1857); ***Placostylus sinistrorsa*** (Crosse, 1884); ***Placostylus souvillei*** (Morelet, 1857); ***Placostylus superfasciatus*** (Gassies, 1871).

***Plectostylus*** Beck, 1837

***Plectostylus broderipii*** (Sowerby I, 1832); ***Plectostylus coturnix*** (Sowerby I, 1832); ***Plectostylus elegans*** (Pfeiffer, 1842); ***Plectostylus elongata*** (d’Orbigny, 1837); ***Plectostylus punctulifer*** (Sowerby I, 1833); ***Plectostylus reflexus*** (Pfeiffer, 1842); ***Plectostylus variegata*** (Pfeiffer, 1842).

***Poecilocharis*** Kobelt, 1891

***Poecilocharis turneri*** (Pfeiffer, 1860).

***Prestonella*** Connolly, 1929

***Prestonella bowkeri*** (Sowerby III, 1890); ***Prestonella nuptialis*** (Melvill and Ponsonby, 1894); ***Prestonella quadingensis*** Connolly, 1929.

***Santacharis*** Iredale, 1927

***Santacharis fuligineus*** (Pfeiffer, 1853).

### Family Odontostomidae Pilsbry and Vanatta, 1894

**Remarks.** We follow here the classification of Simone (2006), with exceptions mentioned below. However, it must be emphasized that a revision of this family, combining morphological and molecular data, is much overdue.

***Anostoma*** Fischer von Waldheim, 1807

***Anostoma brunneum*** Verdcourt, 1990; ***Anostoma carinatum*** Pfeiffer, 1853.

***Bahiensis*** Jousseaume, 1877

***Bahiensis fuscagula*** d’Orbigny, 1837; ***Bahiensis guarani*** (d’Orbigny, 1835); ***Bahiensis longulus*** (Pfeiffer, 1859); ***Bahiensis neglecta*** (Pfeiffer, 1847); ***Bahiensis oblita*** (Reeve, 1848); ***Bahiensis occultus*** (Reeve, 1849); ***Bahiensis paralellus*** (Pfeiffer, 1857); ***Bahiensis rhodinostoma*** (d’Orbigny, 1835).

***Biotocus*** Salgado and Leme, 1990

***Biotocus cumingii*** (Pfeiffer, 1849); ***Biotocus turbinatus*** (Pfeiffer, 1845).

***Bonnanius*** Jousseaume, 1900

***Bonnanius ramagei*** (E.A. Smith, 1890).

***Clessinia*** Döring, 1874

***Clessinia costatus*** (Pfeiffer, 1849).

**Remarks.** Molecular evidence appear to suggest that taxa hitherto placed in ***Spixia*** and ***Clessinia*** belong to the same group (Breure and Romero in press); in case further studies should confirm the synonymy of these taxa, ***Clessinia*** has priority over ***Spixia*** Pilsbry & Vanatta, 1894.

***Cyclodontina*** Beck, 1837

***Cyclodontina catharinae*** (Pfeiffer, 1857); ***Cyclodontina charpentieri*** (Pfeiffer, 1850); ***Cyclodontina corderoi*** Klappenbach, 1958; ***Cyclodontina minor*** (d’Orbigny, 1837).

***Hyperaulax*** Pilsbry, 1897

***Hyperaulax ridleyi*** (E.A. Smith, 1890).

***Moricandia*** Pilsbry & Vanatta, 1898

***Moricandia tolerata*** (Fulton, 1903).

***Odontostomus*** Beck, 1837

***Odontostomus grayanus*** Pfeiffer, 1845; ***Odontostomus odontostomus*** (Sowerby I, 1824).

**Remarks.** Simone (2006) seems to have misinterpreted this genus, by considering ***Plagiodontes daedaleus*** (Deshayes, 1851) as a member of ***Odontostomus***, whilst placing the type species of this genus in ***Macrodontes*** Swainson, 1840 without further discussion.

***Plagiodontes*** Döring, 1876

***Plagiodontes dentata*** (Wood, 1828); ***Plagiodontes patagonica*** (d’Orbigny, 1835).

***Spixia*** Pilsbry & Vanatta, 1894

***Spixia major*** (d’Orbigny, 1837).

### Alphabetic list of taxa by species name

#### Bulimus ouveanus alba

Crosse, 1874

http://species-id.net/wiki/Bulimus_ouveanus_alba

Figs 8G–H

Bulimus ouveanus var. *γ alba*Crosse 1874: 109; Breure and Schouten 1985: 66.
Placostylus fibratus ouveanus (Dotzauser in Mousson); Neubert et al. 2009: 74, fig. 96

##### Type locality.

“in loco Siandé dicto, Novae Caledoniae (*E. Marie*)”.

##### Label.

“Siandé”; label in Crosse’s handwriting glued to the shell.

##### Dimensions.

“Long. 48, diam. maj. 23 1/2 mill.”; holotype H 48.1, D 23.8, W 6.1.

##### Type material.

NHMUK 1929.9.14.6, holotype (Marie coll.).

##### Remarks.

The shell is accompanied by another label in Crosse’s handwriting, stating that on 30 July 1884 he received a message from E. Marie that this shell had to be considered a monstrosity. Neubert et al. (2009) ascribe this taxon to Pfeiffer, but it is unclear if Pfeiffer ever saw this specimen.

##### Current systematic position.

Bothriembryontidae, *Placostylus fibratus ouveanus* (Dotzauer in Mousson, 1869).

#### Bulimus angasianus

Pfeiffer, 1864

http://species-id.net/wiki/Bulimus_angasianus

Figs 2A–B, iii

Bulimus angasianus Pfeiffer 1864: 528; B.J. Smith 1992: 101.
Bothriembryon (Bothriembryon) angasianus (Pfeiffer); Breure 1979: 93.

##### Type locality.

“Port Lincoln, South Australia”.

##### Label.

“Port Lincoln / South Australia”; presumably in Angas’ handwriting.

##### Dimensions.

“Long. 25 1/2, diam. 14 mill.”; syntype H 20.6 D 14.6, W 4.5.

##### Type material.

NHMUK 1870.10.26.176, one syntype (Angas coll.).

##### Remarks.

The label bears “type” in the original handwriting, but the specimen does not match the dimensions given by Pfeiffer. There are three additional syntypes in the Angas collection in the Hancock Museun [now part of the Great North Museum in Newcastle-upon-Tyne] (D. Gordon, pers. comm.)

##### Current systematic position.

Bothriembryontidae, *Bothriembryon angasianus* (Pfeiffer, 1864).

#### Bulimus asopeus

Gassies, 1871

http://species-id.net/wiki/Bulimus_asopeus

Figs 7E, 7v

Bulimus asopeus Gassies 1871: 87; Breure and Schouten 1985: 66; Neubert et al. 2009: 51, 54, fig. 18.

##### Type locality.

[New Caledonia] “Ile des Pins, sud de la Nouvelle-Calédonie”.

##### Label.

No information given. The specimen is marked by Gassies with “T” on the ventral side of the last whorl.

##### Dimensions.

“Long. 50 mill.; diam. maj. 34”; holotype H 49.7, D 35.1, W 6.5.

##### Type material.

NHMUK 1883.11.10.1173, holotype, R.P. Lambert leg. (ex Gassies).

##### Remarks.

The specimen is a monstrosity and was classified as *Placostylus fibratus fibratus* (Martyn, 1784) in a recent revision by Neubert et al. (2009).

##### Current systematic position.

Bothriembryontidae, *Placostylus fibratus fibratus* (Martyn, 1784).

#### Eumecostylus vicinus backhuysi

Delsaerdt, 2010

http://species-id.net/wiki/Eumecostylus_vicinus_backhuysi

Eumecostylus vicinus backhuysi Delsaerdt 2010: 43, pl. 6 figs 10–13.

##### Type locality.

“Marau area, SE Guadalcanal, Solomon Islands”.

##### Label.

“Marau area, SE Guadalcanal, Solomon Islands”.

##### Dimensions.

“68.4 x 29.3 mm”; holotype H 68.4, D 29.3, W 4+.

##### Type material.

NHM 20100568, holotype, R. Moylan leg. (ex Delsaerdt ex Backhuys).

##### Remarks.

The apex of the specimen is missing.

##### Current systematic position.

Bothriembryontidae, *Eumecostylus vicinus backhuysi* Delsaerdt, 2010.

#### Bulimus bairdii

Reeve, 1848

http://species-id.net/wiki/Bulimus_bairdii

Figs 7A, 7i

**Figure 8.**
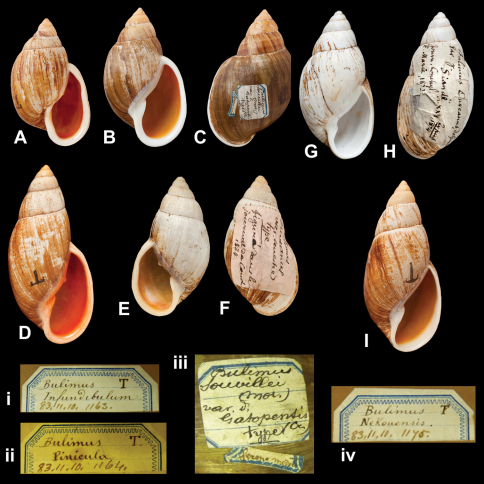
*Placostylus fibratus* subspecies. **A–D** *Placostylus fibratus guestieri* (Gassies, 1869) **A, i** *Bulimus infundibulum* Gassies, 1871, holotype NHM 1883.11.10.1163 (H = 75.6) **B–C, iii** *Bulimus souvillei gatopensis* Crosse, 1870, syntype NHM 1929.9.14.4 (H = 94.5) **D, ii** *Bulimus pinicola* Gassies, 1870, syntype NHM 1883.11.10.1164 (H = 92.8) **E–H** *Placostylus fibratus ouveanus* (Dotzauer in Mousson, 1869) **E–F** *Bulimus ouveanus sinistorsa* Crosse, 1884, NHM 1929.9.14.8 (H = 49.2) **G–H** *Bulimus ouveanus alba* Crosse, 1874, holotype NHM 1929.9.14.6 (H = 48.1) **I, iv** *Placostylus fibratus alexander* (Crosse, 1855), holotype of *Bulimus necouensis* Gassies, 1871 NHM 1883.11.10.1175 (H = 73.9). All reduced in size.

Bulimus bairdii Reeve 1848 [1848–1850]: pl. 43 fig. 272; Breure and Schouten 1985: 67 (lectotype designation); Neubert et al. 2009: 51, 53, fig. 8.

##### Type locality.

“—?”.

##### Label.

No locality given. “Type specimen” added in a later handwriting (E.A. Smith?)

##### Dimensions.

Not given. Specimen figured herein H 78.1, D 36.2, W 7.3.

##### Type material.

NHMUK 1975223, lectotype.

##### Remarks.

Reeve indicated that his specimen was in “Mus. Brit.”. His description leaves room for the possibility that Reeve passed additional specimens to other collectors/institutions; therefore, we concur with Breure and Schouten (1985) to designate this specimen as lectotype. Neubert et al. (2009), who consider this specimen a syntype, treat this taxon as a synonym of *Placostylus fibratus fibratus* (Martyn, 1784).

##### Current systematic position.

Bothriembryontidae, *Placostylus fibratus fibratus* (Martyn, 1784).

#### Bulimus (Mesembrinus?) bowkeri

Sowerby III, 1890

http://species-id.net/wiki/Bulimus_bowkeri

Figs 6A–C, 6i

Bulimus (Mesembrinus?) bowkeri Sowerby III 1890: 581, pl. 56 fig. 5.
Prestonella bowkeri (Sowerby); Herbert 2007: 6, figs 2–7, 9–11, 19, 25 (lectotype designation).

##### Type locality.

[South Africa] “Somerset (East), Cape Colony. Forest about 3,000 ft above sea level (*Col. Bowker*)”.

##### Label.

“Somerset (East), Cape / Colony. Forest about / 3,000 ft above sea level / Col. Bowker”.

##### Dimensions.

“Long. 20, maj. diam. 11 mill.”; lectotype H 19.6, D 10.3, W 4.7.

##### Type material.

NHM 1889.11.4.9, lectotype.

##### Current systematic position.

Bothriembryontidae, *Prestonella bowkeri* (Sowerby III, 1890).

#### Bulimus (Liparus) brazieri

Angas, 1871

http://species-id.net/wiki/Bulimus_brazieri

Figs 2C–E, 2ii

Bulimus (Liparus) brazieri Angas 1871: 19, pl. 1 fig. 28; B.J. Smith 1992: 102.
Bothriembryon (Bothriembryon) brazieri (Angas); Breure 1979: 93.

##### Type locality.

[Western Australia] “Sinclair’s Range, King George’s Sound”; see Remarks.

##### Label.

“ Stirling Range, King George’s Sound”.

##### Dimensions.

“Length 8 lines, breadth 4 lines”; lectotype H 14.3, D 8.7, W 4.9.

##### Type material.

NHMUK 1870.11.5.8, lectotype, and one paralectotype (Angas coll.).

##### Remarks.

According to Iredale (1939) the published locality was in error and should read “Stirling Range”. The taxon forms part of an ill-defined species complex. The material corresponds to the original figures of Angas. The shell pictured in the right-hand view is here selected lectotype (design. n.). The specimens are not full-grown.

##### Current systematic position.

Bothriembryontidae, *Bothriembryon brazieri* (Angas, 1871).

#### Bulinus broderipii

Sowerby I, 1832

http://species-id.net/wiki/Bulinus_broderipii

Figs 4A–B, 4i

Bulinus broderipii Sowerby I in Broderip and Sowerby 1832a: 30; Sowerby 1833 [1833–1838]: figs 1–1*.

##### Type locality.

“prope Copiapo Chilensium”.

##### Label.

“Chili” (in a later handwriting on board). M.C. label style I.

##### Dimensions.

“long. 1 6/8, lat. 1 1/8 poll. [44.3, 28.5 mm]”; lectotype H 44.3, D 26.1, W 5.9.

##### Type material.

NHMUK 20100655, lectotype, and three paralectotypes (Cuming coll.).

##### Remarks.

This taxon may be confused with several other *Plectostylus* species; a revision of this group is pending (F. Cádiz, pers. commun.). One of the specimens matches the original shell height and corresponds to the figures in Sowerby 1833–1838; this shell is here selected as lectotype (design. n.).

##### Current systematic position.

Bothriembryontidae, *Plectostylus broderipii* (Sowerby I, 1832).

#### Anostoma octodentatum brunneum

Verdcourt, 1991

http://species-id.net/wiki/Anostoma_octodentatum_brunneum

Figs 16D–F

Anostoma octodentatum brunneum Verdcourt 1991: 81, fig. 1.
Anostoma brunneum Verdcourt; Simone 2006: 174, fig. 617.

##### Type locality.

“Brazil, Goias State, about 20 km SE. Dianapolis, near Jardim”.

##### Label.

“Brazil, Goiás, near Jardim, c. 20 km SE Dianapolis”.

##### Dimensions.

“Alt. 16 mm, greater diam. 33–34 mm”; holotype H 16.3, D 32.6, W 4.3.

##### Type material.

NHMUK 1990125, holotype and NHMUK 1990126, (juvenile) paratype (R.M. Hartley leg., 12.ii.1987); in ethanol.

##### Remarks.

A paratype in the MZSP collection was figured by Simone (2006).

##### Current systematic position.

Odontostomidae, *Anostoma octodentatum brunneum* Verdcourt, 1991.

#### Bulimus bulbulus

Gassies, 1871

http://species-id.net/wiki/Bulimus_bulbulus

Figs 7F, 7vi

Bulimus bulbulus Gassies 1871: 193.

##### Type locality.

[New Caledonia] “les environs de Nouméa, de Concepcion, Baie du Sud, etc., fossile à l’île des Pins”.

##### Label.

No type locality. Taxon label in Gassies’ handwriting.

##### Dimensions.

“Diam. maj. 4 mill., alt. 5 mill.”; specimen figured herein H 4.13, D 3.56, W 2.1.

##### Type material.

NHMUK 1883.11.10.223-229, seven syntypes (Gassies coll.).

**Remarks.** Three specimens are still glued on board, four others separate in capsule. These specimens are juveniles, probably of *Placostylus fibratus* (Martyn, 1784).

##### Current systematic position.

Bothriembryontidae, *Placostylus cf. fibratus* (Martyn, 1784).

#### Placostylus calus

E.A. Smith, 1891

http://species-id.net/wiki/Placostylus_calus

Figs 11A, 11i

Placostylus calus E.A. Smith 1891: 489, pl. 40 fig. 7.
Placostylus (Placocharis) calus (E.A. Smith) [sic, no parenthesis required]; Breure and Schouten 1985: 63 (lectotype designation).
Eumecostylus calus (E.A. Smith); Delsaerdt 2010: 44, pl. 7 figs 1–4.

##### Type locality.

“Solomon Islands”.

##### Label.

“Solomon Is.”, in Smith’s handwriting.

##### Dimensions.

“Longit. 78 millim., diam. maj. 33”; lectotype H 78, D 33.7, W 5+.

##### Type material.

NHMUK 1891.11.3.4, lectotype; NHMUK1891.11.3.5-6, two paralectotypes (ex Brazier).

##### Remarks.

The apex of the lectotype is damaged.

##### Current systematic position.

Bothriembryontidae, *Eumecostylus calus* (E.A. Smith, 1891).

#### Anostoma carinatum

Pfeiffer, 1853

http://species-id.net/wiki/Anostoma_carinatum

Figs 16A–C, 16i

Anostoma carinatum Pfeiffer 1853a: 57.
Ringicella carinata (Pfeiffer); Simone 2006: 175, fig. 622.

##### Type locality.

“Brasilia (*Mus. Dennison*.)”.

##### Label.

“Brazils”.

##### Dimensions.

“Diam. maj. 24 1/2, (..) alt. 13 mill.”; specimen figured herein H 13.3, D 24.3, W 4.9.

##### Type material.

NHMUK 20100509, three syntypes (Cuming coll.).

**Remarks.** Although the taxon label is not in Pfeiffer’s handwriting, the specimens fit the original measurements and are considered probable syntypes.

##### Current systematic position.

Odontostomidae, *Anostoma carinatum* Pfeiffer, 1853.

#### Bulimus catharinae

Pfeiffer, 1857

http://species-id.net/wiki/Bulimus_catharinae

Figs 23A–B, 23i

Bulimus catharinae Pfeiffer 1857: 389; Breure 1974: 113.
Cyclodontina (Cyclodontina) fusiformis (Menke); Breure and Schouten 1985: 6, pl. 1 figs 1–2 (lectotype designation).
Cyclodontina catharinae (Pfeiffer); Simone 2006: 165, fig. 567.

##### Type locality.

“St. Catherine’s, Brazil”.

##### Label.

“St Catherines Brazil”; taxon label in Pfeiffer’s handwriting.

##### Dimensions.

“23–25, 7 1/2–8 mill.”; specimen figured herein H 24.4, D 7.5, W 8.1.

##### Type material.

NHMUK 1975261, lectotype and two paralectotypes (Cuming coll.).

##### Remarks.

Simone revalidated this taxon without providing further discussion; however, we follow here the synonymization of Breure and Schouten (1985).

##### Current systematic position.

Odontostomidae, *Cyclodontina fusiformis* (Menke, 1828).

#### Bulimus charpentieri

Pfeiffer, 1850

http://species-id.net/wiki/Bulimus_charpentieri

Figs 21G–I, 21ii

Bulimus charpentieri “Grateloup” Pfeiffer 1850: 14; Pfeiffer in Küster and Pfeiffer 1854 [1840–1865]: 143, pl. 45 figs 14–15; Breure 1974: 113.

##### Type locality.

“Cardova [sic, Córdoba] reipubl. Argentinae”.

##### Label.

“Brazils”. M.C. label style III.

##### Dimensions.

“Long. 20, diam. 5 2/3 mill.”; specimen figured herein H 17.9, D 5.15, W 9.2.

##### Type material.

NHMUK 20110386, syntype (Cuming coll.).

##### Current systematic position.

Odontostomidae, *Cyclodontina charpentieri* (Pfeiffer, 1850).

#### Cyclodontina (Spixia) corderoi

Klappenbach, 1958

http://species-id.net/wiki/Cyclodontina_corderoi

Figs 21J–K, 21iii

Cyclodontina (Spixia) corderoi Klappenbach 1958: 2, pls 1–2; Breure 1974: 114.

##### Type locality.

[Uruguay] “Pozo Hondo, próxima a la población Tambores en el departemento de Tacuarembó”.

##### Label.

“Pozo Hondo, Tambores, Depto Tacuarembó, Rep. del Uruguay”.

##### Dimensions.

“Largo total 17.5, Ancho 5 (en millimetros)”; specimen figured herein H 16.1, D 5.44, W 7+.

##### Type material.

NHMUK 1961679, three paratypes, M.A. Klappenbach & P. San Martin leg., 12.x.1957, ex MNHNM 0716.

##### Remarks.

The apex of the shell is slightly damaged.

##### Current systematic position.

Odontostomidae, *Cyclodontina corderoi* Klappenbach, 1958.

#### Bulimus corpulentus

Gassies, 1871

http://species-id.net/wiki/Bulimus_corpulentus

Figs 10A, 10i

Bulimus corpulentus Gassies 1871: 183; Breure and Schouten 1985: 67.
Placostylus corpulentus (Gassies); Neubert et al. 2009: 112, fig. 184.

##### Type locality.

“Fossile à l’île des Pins et à l’îlot Koutoumo”.

##### Label.

“New Caledonia”.

##### Dimensions.

“Long. 100 mill.; diam. maj. 55 mill.”; specimen figured herein H 94.0, D 64.0, W 6.7.

##### Type material.

NHMUK 1883.11.10.1178, syntype, R.P. Lambert leg. (ex Gassies).

##### Remarks.

The surface of the specimen is covered by various encrustations.

##### Current systematic position.

Bothriembryontidae, *Placostylus corpulentus* (Gassies, 1871).

#### Bulimus costatus

Pfeiffer, 1848

http://species-id.net/wiki/Bulimus_costatus

Figs 22F–G, 22ii

Bulimus costatus Pfeiffer 1848: 114; Pfeiffer 1849a: 111; Reeve 1849 [1848–1850]: pl. 65 fig. 450.
Clessinia costata (Pfeiffer); Breure and Schouten 1985: 10, pl. 1 fig. 4 (lectotype designation); Simone 2006: 164, fig. 563.

##### Type locality.

“Brasilia” [Brazil].

##### Label.

“Brazil”. M.C. label style III. Another label indicates that this specimen was also figured in Reeve’s Conchologica Iconica.

##### Dimensions.

“Long. 18, diam. 5 1/2 mill.”; lectotype H 17.8, D 5.6, W 8.8.

##### Type material.

NHMUK 1975250, lectotype and two paralectotypes (Cuming coll.).

##### Remarks.

Although the first publication was in Pfeiffer (1848), he refers to Pfeiffer (1849) as the original description; this paper was describing new species from the Cuming collection.

##### Current systematic position.

Odontostomidae, *Clessinia costata* (Pfeiffer, 1848).

#### Bulinus coturnix

Sowerby I, 1832

http://species-id.net/wiki/Bulinus_coturnix

Figs 4C–D, 4ii

Bulinus coturnix Sowerby I in Broderip and Sowerby 1832a: 30.

##### Type locality.

[Chile] “Huasco”.

##### Label.

“Huasco”. M.C. label style I.

##### Dimensions.

“Long 1 1/4, lat. 7/8 poll. [H 31.7, D 22.2 mm]”; specimen figured herein H 32.4, D 21.0, W 5.0.

##### Type material.

NHMUK 20100620, five possible syntypes (Cuming coll.).

##### Remarks.

Of the five specimens, only one is adult; although it does not correspond to the original measurements, it originates from the type locality. The material is considered as possible syntypes.

##### Current systematic position.

Bothriembryontidae, *Plectostylus coturnix* (Sowerby I, 1832).

#### Bulimus crassilabrum

Garrett, 1872

http://species-id.net/wiki/Bulimus_crassilabrum

Figs 13A, 13i

Bulimus crassilabrum Garrett 1872: 233, pl. 18 fig. 5.

##### Type locality.

[Fiji] “Vanna Levu Id., Viti Isles”.

##### Label.

“Vanua Levu”.

##### Dimensions.

“Length 41 mill., diam. 21 mill.”; specimen figured herein H 44.0, D 22.1, W 5.1.

##### Type material.

NHMUK 20030801, one syntype, ex Kennard ex Garrett coll. (“cotype ex auct.”).

##### Remarks.

Richardson (1995: 286) placed this taxon in the synonymy of *Aspastus rugatus* (Garrett, 1872), without further comments. Judging by the descriptions and figures of both species, there is every reason to consider these two taxa identical. Based on Garrett’s 1872 paper, *Bulimus crassilabrum* has page priority.

##### Current systematic position.

Bothriembryontidae, *Callistocharis crassilabrum* (Garrett, 1872).

#### Tomigerus cumingii

‘Newcomb’ Pfeiffer, 1849

http://species-id.net/wiki/Tomigerus_cumingii

Figs 19A–C, 19i

Tomigerus cumingii ‘Newcomb’ Pfeiffer 1849b: 67.
Biotocus cumingii (Pfeiffer); Simone 2006: 177, fig. 633.

##### Type locality.

“Para[,] Brasiliae”.

##### Label.

“Para”. M.C. label style III.

##### Dimensions.

“Diam. maj. 7 1/2, min. 5, alt. 6 mill.”; specimen figured herein H 6.45, D 6.72, W 4.8.

##### Type material.

NHMUK 1975587, three syntypes, ex Newcomb (Cuming coll.).

##### Current systematic position.

Odontostomidae, *Biotocus cumingii* (‘Newcomb’ Pfeiffer, 1849).

#### Bulimus cuninculinsulae

Cox, 1872

http://species-id.net/wiki/Bulimus_cuninculinsulae

Figs 12C, 12iii

Bulimus cuninculinsulae Cox 1872b: 19, 233, pl. 18 fig. 5; Breure and Schouten 1985: 68 (lectotype designation).

##### Type locality.

[Australia] “Rabbit Island, near Lord Howe’s Island, Pacific Ocean”.

##### Label.

“Rabbit Is. / near Lord / Howe’s Is. / Pacific ocean”.

##### Dimensions.

“Length 1.65, breadth 0.75 of an inch [H 41.9, D 19.1 mm]”; lectotype H 41.6, D 20.5, W 5.6.

##### Type material.

NHMUK 1880.12.11.47-48, lectotype and one paralectotype (ex Cox).

##### Remarks.

The specimen figured by Cox (1872) has been chosen by Breure and Schouten (1985) as the lectotype; the paralectotype matches the original measurements (H 41.9).

##### Current systematic position.

Bothriembryontidae, *Placostylus cuninculinsulae* (Cox, 1872).

#### Placostylus (Euplacostylus) cylindricus

Fulton, 1907

http://species-id.net/wiki/Placostylus_cylindricus

Figs 11C, 11iii

Placostylus (Euplacostylus) cylindricus Fulton 1907: 154, pl. 10 fig. 3.
Placostylus (?Proaspastus) cylindricus (Fulton); Breure and Schouten 1985: 64.
Eumecostylus cylindricus (Fulton); Delsaerdt 2010: 39, pl. 5 figs 1–5.

##### Type locality.

“Isabel Island, Solomons” (see remarks).

##### Label.

“Isabel Id. / Solomon Ids.”, in Fulton’s handwriting.

##### Dimensions.

“Maj. diam. 23, alt. 71 mm.”; lectotype H 71.3, D 27.4, W 6.7.

##### Type material.

NHMUK 1907.5.3.160, lectotype (ex Fulton).

##### Remarks.

Fulton (1907) mentioned that he had three specimens, of which the NHMUK specimen corresponds to his figure. Contrary to Breure and Schouten (1985), it should not be considered as holotype, but is here designated as the lectotype (Art. 74.6 ICZN). The specimen has the apex damaged. Clench (1941) questioned the type locality and suggested it was erroneous. Also Delsaerdt (2010) has been unable to locate this species on Santa Isabel; it is only known from Guadalcanal.

##### Current systematic position.

Bothriembryontidae, *Eumecostylus cylindricus* (Fulton, 1907).

#### Pupa dentata

Wood, 1828

http://species-id.net/wiki/Pupa_dentata

Figs 25A–D, 25i

Pupa dentata Wood 1828: 24, 50, pl. 8 fig. 71.

##### Type locality.

Not given.

##### Label.

“Montevideo”.

##### Dimensions.

Not given. Specimen figured herein H 14.16, D 7.17, W 7.9.

##### Type material.

NHMUK 1840.9.12.50, two syntypes, ex Mrs. Mawe.

##### Remarks.

Wood (1828: 24) referred to material from “Br.M. [British Museum]”. This supports the claim expressed on the label that this material may be considered as probable syntypes. It is not clear, however, on what evidence the locality “Montevideo” is based, as Wood did not state it in his publication. One specimen is damaged.

##### Current systematic position.

Odontostomidae, *Plagiodontes dentata* (Wood, 1828).

#### Bulimus dux

Pfeiffer, 1861

http://species-id.net/wiki/Bulimus_dux

Figs 3A–B, 3i

Bulimus dux Pfeiffer 1861: 24; B.J. Smith 1992: 103.
Bothriembryon (Bothriembryon) dux (Pfeiffer); Breure 1978: 203 (lectotype designation); Breure 1979: 94.

##### Type locality.

“King George’s Sound, [Western] Australia”.

##### Label.

“King Georges Sound / W. Australia”; taxon label damaged, possibly in Pfeiffer’s handwriting. M.C. label style IV.

##### Dimensions.

“Long. 51, diam. 26 mill.”; lectotype H 48.5, D 28, W 6.0.

**Type material.** NHMUK 19598, lectotype; 19599, two paralectotypes (Cuming coll.).

##### Remarks.

These specimens were identified by E.A. Smith as type material.

##### Current systematic position.

Bothriembryontidae, *Bothriembryon dux* (Pfeiffer, 1861).

#### Bulimus eddystonensis

Pfeiffer, 1855

http://species-id.net/wiki/Bulimus_eddystonensis

Figs 9D, 9iv

Bulimus eddystonensis Pfeiffer 1855a: 8.
Placostylus (Placostylus) eddystonensis (Pfeiffer); Breure and Schouten 1985: 59 (lectotype designation); Neubert et al. 2009: 94, fig. 149.

##### Type locality.

“Eddystone Island, Australian Seas” [Solomon Islands; see remarks].

##### Label.

“Eddystone Isld / Australian sea”, taxon label in Pfeiffer’s handwriting. M.C. label style IV.

##### Dimensions.

“Long. 74, diam. 34 mill.”; lectotype H 73.3, D 36.3, W 6.2.

##### Type material.

NHMUK 1975230, lectotype and two paralectotypes (Cuming coll.).

**Remarks.** The type locality was in error and was subsequently corrected to New Caledonia, Hienghène (Gassies 1863: 260); see Neubert et al. 2009: fig. 156 for distributional data.

##### Current systematic position.

Bothriembryontidae, *Placostylus eddystonensis* (Pfeiffer, 1855).

#### Bulimus edwardsianus

Gassies, 1863

http://species-id.net/wiki/Bulimus_edwardsianus

Figs 9F, 9vi

Bulimus edwardsianus Gassies 1863: 40, pl. 4 fig. 2; Breure and Schouten 1985: 60.
Placostylus caledonicus (Petit); Neubert et al. 2009: 104, fig. 171.

##### Type locality.

[New Caledonia] “Nékété, Kanala, l’île Nu, l’île Pôt”.

##### Label.

“Nékété”, taxon label in Gassies’ handwriting, marked “T”.

##### Dimensions.

“Long. 80 mill.—Diam. maj. 34 mill.”; specimen figured herein H 72.0, D 36.5, W 6.7.

##### Type material.

NHMUK 1883.11.10.1172, one possible syntype (ex Gassies ex Marie).

##### Remarks.

Neubert et al. (2009) consider this specimen a possible syntype; it is not the originally figured specimen.

##### Current systematic position.

Bothriembryontidae, *Placostylus caledonicus* (Petit de la Saussaye, 1845).

#### Succinea elegans

Pfeiffer, 1842

http://species-id.net/wiki/Succinea_elegans

Figs 4G–H, 4iv

Succinea elegans Pfeiffer 1842: 56; Pfeiffer 1843: 187; Pfeiffer 1855, in Küster and Pfeiffer 1840–1865: pl. 60 fig. 7. Not *Succinea elegans* Risso, 1826.
Plectostylus coquimbensis perelegans (Pilsbry); Breure 1978: 201, pl. 9 fig. 14 (lectotype designation).

##### Type locality.

“Huasco, Chile (*Bridges*)”.

##### Label.

“Questa de Arenas / Huasco, Chili”, taxon label in Pfeiffer’s handwriting. M.C. label style IV.

##### Dimensions.

“Long. 36, diam. 17 mill.”; lectotype H 30.0, D 16.2, W 5.1.

##### Type material.

NHMUK 1975360, lectotype; 1975361, three paralectotypes (Cuming coll.).

##### Remarks.

The lectotype corresponds to the specimen figured by Pfeiffer (1855 [1840–1865]). The original shell height may have been in error. This taxon is now considered a junior subjective synonym of *Bulinus broderipii* Sowerby I, 1832 (F. Cádiz, unpublished data).

##### Current systematic position.

Bothriembryontidae, *Plectostylus broderipii* (Sowerby I, 1832) (synon. n.).

#### Bulimus broderipi elongata

d’Orbigny, 1837

http://species-id.net/wiki/Bulimus_broderipi_elongata

Figs 4E–F, 4iii

Bulimus broderipi var. *elongata*d’Orbigny 1837 [1834–1847]: 266.

##### Type locality.

“Cobija, Bolivia” [Chile].

##### Label.

“Cobija”; in d’Orbigny’s handwriting.

##### Dimensions.

“Long. 30 millim.; lat. 17 millim.”; lectotype H 30.5, D 16.4, W 5.2.

##### Type material.

NHMUK 1854.12.4.102, lectotype and six paralectotypes (d’Orbigny coll.).

##### Remarks.

No material of this taxon has been found in the MNHN. As the species was not figured by d’Orbigny, the specimen that most closely matches the given dimensions has been chosen as the lectotype. This taxon is now considered a junior subjective synonym of *Bulinus broderipii* Sowerby I, 1832 (F. Cádiz, unpublished data).

##### Current systematic position.

Bothriembryontidae, *Plectostylus broderipii* (Sowerby I, 1832) (synon. n.).

#### Bulimus falcicula

Gassies, 1871

http://species-id.net/wiki/Bulimus_falcicula

Figs 7B, 7ii

Bulimus falcicula Gassies 1871: 190.
Placostylus (Placostylus) falcicula (Gassies); Breure and Schouten 1985: 59.
Placostylus (Placostylus) fibratus fibratus (Martyn); Neubert et al. 2009: 50, fig. 15.

##### Type locality.

[New Caledonia] “Baie du Sud (Nouvelle-Calédonie)”.

##### Label.

No locality label; taxon label in Gassies’ handwriting.

##### Dimensions.

“Long. 80 mill.; diam. maj. 40 mill.”; specimen figured herein H 74.5, D 35.9, W 6.5.

##### Type material.

NHMUK 1883.11.10.1174, syntype, R.P. Lambert leg. (ex Gassies).

##### Remarks.

The taxon label is marked “T”, and the shell is marked with “245” on the last whorl.

##### Current systematic position.

Bothriembryontidae, *Placostylus fibratus fibratus* (Martyn, 1784).

#### Placostylus (Eumecostylus) foxi

Clench, 1950

http://species-id.net/wiki/Placostylus_foxi

Figs 11B, 11ii

Placostylus (Eumecostylus) foxi Clench 1950: 3, figs 1–2; Breure and Schouten 1985: 68.
Eumecostylus foxi Clench; Delsaerdt 2010: 34, pl. 3 figs 9–15.

##### Type locality.

“Heuru western end of San Cristobal Island, Solomon Islands”.

##### Label.

“Brit. Solomon Isl. / Heuru, western Cristobal Isl.”.

##### Dimensions.

“Length 86.8, Gt. width 32 [mm]”; specimen figured herein H 82.3, D 31.5, W 5.9.

##### Type material.

NHMUK 1953.2.4.1, paratype (ex Schlesch).

##### Remarks.

Delsaerdt (2010) corrected the coordinates of the type locality as given by Clench. The specimen in NHMUK was obtained “Through Dr Quick” from the Schlesch collection and has a label “paratype” glued within the aperture. It may be noted that Clench (1950) stated “Paratypes (...) in the Museum of Comparative Zoology, the B.P. Bishop Museum and the collections of A.G. Stevenson, Reverend Fox and Walter J. Eyerdam”. It is not clear how the specimen reached the Schlesch collection or if it was genuinely part of the original series.

##### Current systematic position.

Bothriembryontidae, *Eumecostylus foxi* (Clench, 1950).

#### Bulimus fuligineus

Pfeiffer, 1853

http://species-id.net/wiki/Bulimus_fuligineus

Figs 15A, 15i

Bulimus fuligineus Pfeiffer 1853b: 301; Pfeiffer 1854, in Küster and Pfeiffer 1840–1865: 157, pl. 48 figs 5–6.
Bulimus fuligineus var. β Pfeiffer 1859: 363.
Placostylus (Santacharis) fuligineus (Pfeiffer); Solem 1959: 134, pl. 8 figs 3, 6.

##### Type locality.

[Vanuatu] “Novis Hebridis”.

##### Label.

“New Hebrides”. “Isle of Aneiteum”; taxon labels in Pfeiffer’s handwriting. M.C. label style IV.

##### Dimensions.

“Long. 38, diam. 16 mill.”; lectotype H 39.4, D 19.2, W 4.6.

##### Type material.

NHMUK 1975226, lectotype and two paralectotypes; 20100650, three paralectotypes of the varietal form, all Macgillivray leg. (Cuming coll.).

##### Remarks.

Two lots, each consisting of three specimens proved to be present, one corresponding to the variety recognized by Pfeiffer (1859). All material was taken at Vanuatu, Prov. Tafea, Anatom (of which Anaiteum and Aneityum are a few of the variant names), according to the label glued inside the aperture of one of the specimens and the label of the variety lot. The specimen figured by Pfeiffer (1854: pl. 48 figs 5–6) is now seclected lectotype (design. n.).

##### Current systematic position.

Bothriembryontidae, *Santacharis fuligineus* (Pfeiffer, 1853).

#### Bulimus fuscagula

d’Orbigny, 1837

http://species-id.net/wiki/Bulimus_fuscagula

Figs 23E–F, 23iii

Bulimus fuscagula d’Orbigny 1837 [1834–1847]: 318, pl. 39 figs 1–2; Gray 1854: 22.
Bahiensis miliola (d’Orbigny); Simone 2006: 169, fig. 591b.

##### Type locality.

[Brazil] “Rio de Janeiro”.

##### Label.

“In Bresil”

##### Dimensions.

“Long. 30 millim.; lat. 8 millim.”; lectotype H 28.6, D 7.6, W 9.4.

##### Type material.

NHMUK 1854.12.4.141, lectotype (d’Orbigny coll.).

##### Remarks.

This specimen was thought by d’Orbigny to represent *Auricula fuscagula* Lea, 1837 [= *Cyclodontina punctatissima* (Lesson, 1830)], which is, however, a distinct species. The type lot consisted of a second specimen (NHMUK 1854.12.4.142) which indeed belongs to this species described by Lesson. Therefore, to solve the potential confusion, specimen NHMUK 1854.12.4.141 is here designated as lectotype (design. n.) of *Bulimus fuscagula* d’Orbigny.

##### Current systematic position.

Odontostomidae, *Bahiensis fuscagula* (d’Orbigny, 1837).

#### Bulimus souvillei gatopensis

Crosse, 1870

http://species-id.net/wiki/Bulimus_souvillei_gatopensis

Figs 8B–C, 8iii

Bulimus souvillei var. δ *gatopensis*Crosse 1870: 242; Breure and Schouten 1985: 69; Neubert et al. 2009: 61.

##### Type locality.

“Gatope et in loco ‘Ferme modèle’ dicto, Novae Caledoniae. (E. Marie)”.

##### Label.

No locality given. Taxon label in Crosse’s handwriting (?).

##### Dimensions.

“Long. 88–95, diam. maj. 47–56 mill.”; specimen figured herein H 94.5, D 55.5, W 6.5.

##### Type material.

NHMUK 1929.9.14.4, syntype, ex Marie coll.

##### Remarks.

A label (from Crosse?) with the note “type” is glued on the dorsal side of the last whorl. Neubert et al. (2009) have commented on the type locality of this material, noting it to be erroneous or based on introduced material. A further syntype is in the MNHN collection.

**Current systematic position.** Bothriembryontidae, *Placostylus fibratus guestieri* (Gassies, 1869).

#### Bulimus fibratus grammica

Crosse, 1870

http://species-id.net/wiki/Bulimus_fibratus_grammica

Figs 9A, 9i

Bulimus fibratus grammica Crosse 1870: 242; Neubert et al. 2009: 62, fig. 54.

##### Type locality.

“Nouvelle-Calédonie”.

##### Label.

“New Caledonia” added in a later handwriting. “type de l’auteur” glued on shell.

##### Dimensions.

“Long. 111, diam. maj. 47 mill.”; specimen figured herein H 111.3, D 52.5, W 7.4.

##### Type material.

NHMUK 1929.9.14.5, syntype, ex Marie coll.

##### Remarks.

In the recent revision of New Caledonian *Placostylus* (Neubert et al. 2009) an argument is made for the current systematic position of this taxon.

##### Current systematic position.

Bothriembryontidae, *Placostylus fibratus goroensis* (Souverbie, 1870).

#### Bulimus grayanus

Pfeiffer, 1845

http://species-id.net/wiki/Bulimus_grayanus

Figs 24E–H, 24ii

Bulimus grayanus Pfeiffer 1845b: 73.
Macrodontes grayanus (Pfeiffer); Simone 2006: 162, fig. 556.

##### Type locality.

“Brazils”.

##### Label.

“Brazil”. M.C. label style III.

##### Dimensions.

“Long. 35, diam. 11 mill.”; specimen figured herein H 34.1, D 12.8, W 6.0.

##### Type material.

NHMUK 20110387, syntype (Cuming coll.).

##### Current systematic position.

Odontostomidae, *Odontostomus grayanus* (Pfeiffer, 1845).

#### Helix guarani

d’Orbigny, 1835

http://species-id.net/wiki/Helix_guarani

Figs 18A–B, 18iii

Helix guarani d’Orbigny 1835: 21.
Bulimus guarani d’Orbigny 1837 [1834–1847]: 518, pl. 41bis fig. 1.

##### Type locality.

“provincia Corrientesensi (republica Argentina)”.

##### Label.

“corrientes. rep. Argentina”, label in d’Orbigny’s handwriting.

##### Dimensions.

“Longit. 23 millim., latit. 8 1/2”; lectotype H 20.5, D 7.4, H 8.1.

##### Type material.

NHMUK 1854.12.4.140, lectotype and one paralectotype (d’Orbigny coll.).

##### Remarks.

The specimen corresponding to d’Orbigny 1837 [1834–1847]: pl. 41 bis fig. 1 has been selected lectotype (design. n.); the apex of the specimen is slightly damaged and its current shell height is somewhat smaller than in the original measurements. This species is tentatively classified with *Bahiensis*, awaiting a generic level revision of this group.

##### Current systematic position.

Odontostomidae, *Bahiensis guarani* (d’Orbigny, 1835).

#### Placostylus guppyi

E.A. Smith, 1891

http://species-id.net/wiki/Placostylus_guppyi

Figs 14C, 14iii

Placostylus guppyi E.A. Smith 1891: 489, pl. 40 fig. 6; Breure and Schouten 1985: 69 (lectotype designation).
Placocharis guppyi (Smith); Delsaerdt 2010: 60, pl. 11 figs 12–13.

##### Type locality.

“Solomon Islands”; restricted to West Guadalcanal by Delsaerdt (2010).

##### Label.

“Solomon Is.”, taxon label in Smith’s handwriting.

##### Dimensions.

“Longit. 80 millim., diam. maj. 37”; lectotype H 80.1, D 40.1, W 5.5.

##### Type material.

NHMUK 1891.11.6.110, lectotype; 1891.11.6.111, paralectotype, Brazier leg.

##### Current systematic position.

Bothriembryontidae, *Placocharis guppyi* (E.A. Smith, 1891).

#### Bulimus hargravesi

Cox, 1871

http://species-id.net/wiki/Bulimus_hargravesi

Figs 12A, 12i

Bulimus hargravesi Cox 1871: 323, pl. 34 fig. 3; Breure and Schouten 1985: 69.
Eumecostylus hargravesi (Cox); Delsaerdt 2010: 47, pl. 8 figs 1–6.

##### Type locality.

“Treasury Island, Solomon Islands”, corrected to Malaita, Solomon Islands (Delsaerdt 2010).

##### Label.

“Solomon Isls”.

##### Dimensions.

“Diam. 1.05, long. 2.60 unc. [H 64.3, D 25.9 mm]”; lectotype H 65.0, D 30.9, W 5.3.

##### Type material.

NHMUK 1880.12.11.43, lectotype (ex Cox).

##### Remarks.

The holotype designation (Breure and Schouten 1985) has to be interpreted as lectotype designation (Art. 74.6 ICZN); the claim of Delsaerdt (2010: unnumbered page opposite contents [not repeated in main text]) of a lectotype designation is thus erroneous. Delsaerdt interpreted Cox’ abbreviation “unc.” equalling 25.4 mm given his dimensions of the type specimens as 26.67 x 66.04 mm. Cox’ abbreviation can also be read as the Roman uncia, equal to 2.473 cm (Rowlett 2004), but according to Woodward (1851 [1851–1856]: 61) the uncia equals the inch (= 25.4 mm).

##### Current systematic position.

Bothriembryontidae, *Eumecostylus hargravesi* (Cox, 1871).

#### Bulimus infundibulum

Gassies, 1871

http://species-id.net/wiki/Bulimus_infundibulum

Figs 8A, 8i

Bulimus infundibulum Gassies 1871: 86; Breure and Schouten 1985: 69; Neubert et al. 2009: 55, fig. 23.

##### Type locality.

[New Caledonia] “Ilôt Koutoumo, sud de la Nouvelle-Calédonie”.

##### Label.

No locality label. Taxon label in Gassies’ handwriting and marked “T”.

##### Dimensions.

“Long. 75 mill.; diam. maj. 40”; holotype H 75.6, D 46, W 6.5.

##### Type material.

NHMUK 1883.11.10.1163, holotype, ex Gassies.

##### Remarks.

Gassies (1871) remarked that he had seen only one specimen. The single specimen known is a monstrosity; it is marked “241” on the last whorl.

##### Current systematic position.

Bothriembryontidae, *Placostylus fibratus guestieri* (Gassies, 1869)

#### Bulimus kingii

J.E. Gray, 1825

http://species-id.net/wiki/Bulimus_kingii

Figs 2F–G, 2i

Bulimus kingii J.E. Gray 1825: 414; B.J. Smith 1992: 104.
Bothriembryon (Bothriembryon) kingii (Gray); Breure 1978: 205 (lectotype designation); Breure 1979: 94.

##### Type locality.

[Australia] “New Holland, *Capt. King*”.

##### Label. 

“Australia”.

##### Dimensions.

“Long. 1, diam. 1/2 unc.”; lectotype H 24.5, D 10.6, W 5.5.

##### Type material.

NHMUK 195910, lectotype; 195911, one paralectotype, ex P.P. King R.N.

##### Remarks.

According to Pilsbry (1900) and Iredale (1939) the type locality is Western Australia, King George Sound, near Bald Head. Captain King visited this locality on 20 Juanuary 1818 and stated “An abundance of shells of the *helix* tribe (*Helix bulimus*) was found on the top and sides of the hill” (King 1827: 12).

##### Current systematic position.

Bothriembryontidae, *Bothriembryon kingii* (J.E. Gray, 1825).

#### Bulimus koroensis

Garrett, 1872

http://species-id.net/wiki/Bulimus_koroensis

Figs 13E, 13iv

Bulimus koroensis Garrett 1872: 236, pl. 18 fig. 9.

##### Type locality.

“Koro Isl., Viti Isles”.

##### Label.

“Koro, Viti”.

##### Dimensions.

“Length 53 mill.; diam. 18 mill.”; specimen figured herein H 51.2, D 18.0, W 5.1.

##### Type material.

NHMUK 20030802, two probable syntypes, ex Taylor, ex Garrett.

##### Current systematic position.

Bothriembryontidae, *Euplacostylus koroensis* (Garrett, 1872).

#### Bulimus (Charis) kreftii

Cox, 1872

http://species-id.net/wiki/Bulimus_kreftii

Figs 14A, 14i–ii

Bulimus (Charis) kreftii Cox 1872b: 19, pl. 4 fig. 4.
Placocharis kreftii (Cox); Delsaerdt 2010: 67, pl. 11 figs 14–17 (lectotype designation).

##### Type locality.

“Solomon Islands”, restricted to Florida Islands by Delsaerdt (2010).

##### Label.

“Solomon Isles”, in Cox’ handwriting (see remarks).

##### Dimensions.

“Length 2.10, breadth 0.92 of an inch [H 53.3, D 23.3]”; specimen figured herein H 54.9, D 24.3, W 5.2.

##### Type material.

NHMUK 20070068, lectotype and three paralectotypes, ex Kennard ex Cox; 1880.12.11.46, paralectotype, ex Cox.

##### Remarks.

**Figure 14.**
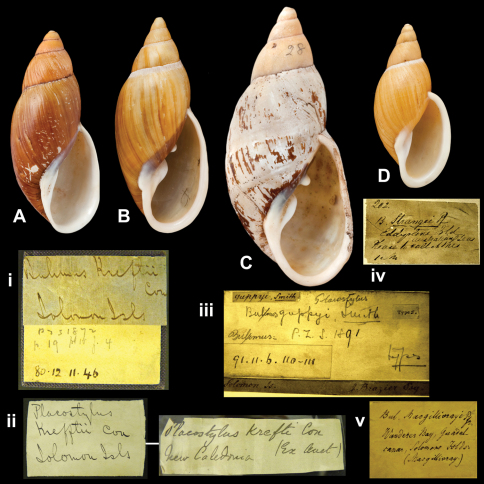
*Placocharis* species. **A, i–ii** *Placocharis kreftii* (Cox, 1872), lectotype NHM 20070068 (H = 54.9) **i** Label of the specimen presented by J.C. Cox **ii** Labels of the specimens originating from the Kennard collection **B, v** *Placocharis macgillivrayi* (Pfeiffer, 1855), lectotype NHM 1975232 (H = 58.5) **C, iii** *Placocharis guppyi* (E.A. Smith, 1891), lectotype NHM 1891.11.6.110 (H = 80.1) **D, iv** *Placocharis strangei* (Pfeiffer, 1853), lectotype NHM 20100652 (H = 45.9).

Both series are accompanied by a label in Cox’ handwriting. The specimen registered 1880.12.11.46 was presented by J.C. Cox, has a taxon label “Bulimus kreftii” and corresponds to pl.4 fig. 4 of Cox (1872). The other lot (ex A.S. Kennard collection) has a taxon label “Placostylus krefftii” [sic, misspelled] and is “(Ex Auct.)”; the locality on Kennard’s label “New Caledonia” is clearly in error. It is therefore more likely that the specimen presented by Cox was truly part of the type series; those from the Kennard collection may have been collected at a later stage, for which the genus name *Placostylus* on the taxon label written by Cox (Fig. 14ii) is an indication.

##### Current systematic position.

Bothriembryontidae, *Placocharis kreftii* (Cox, 1872).

#### Bulimus leeuwinensis

E.A. Smith, 1894

http://species-id.net/wiki/Bulimus_leeuwinensis

Figs 3C–E, 3ii

Bulimus leeuwinensis E.A. Smith 1894: 94, pl. 7 fig. 27; B.J. Smith 1992: 105.
Bothriembryon (Bothriembryon) leeuwinensis (Smith); Breure 1978: 206 (lectotype designation); Breure 1979: 94.

##### Type locality.

“Cape Leeuwin, S.W. Australia”.

##### Label.

“Cape Leeuwin, S.W. Australia”.

##### Dimensions.

“Longit. 27 mm.; diam. 12.5 mm.”; lectotype H 27, D 13.7, W 5.4.

##### Type material.

NHMUK 1891.11.21.128, lectotype; 1891.11.21.129–132, four paralectotypes, J.J. Walker leg.

##### Current systematic position.

Bothriembryontidae, *Bothriembryon leeuwinensis* (E.A. Smith, 1894).

#### Bulimus longulus

‘Behn’ Pfeiffer, 1859

http://species-id.net/wiki/Bulimus_longulus

Figs 17A–B, 17i

Bulimus longulus ‘Behn’ Pfeiffer 1859a: 44.
Bahiensis longulus (Pfeiffer); Simone 2006: 169, fig. 590.

##### Type locality.

[Brazil] “Chicatas Brasiliae”.

##### Label.

“Brazils”. M.C. label style III.

##### Dimensions.

“Long. 30, diam. 7 1/2 mill.”; specimen figured herein H 29.8, D 7.23, W 11.2.

##### Type material.

NHMUK 20110388, syntype (Cuming coll.).

##### Remarks.

Pfeiffer (1859) stated that the material was in “Mus. Cuming”. The specimens found are not accompanied by a taxon label in Pfeiffer’s handwriting, but their type status is not disputed. Simone (2006) listed the specimen as lectotype, but we are unaware of a previous lectotype designation for this taxon. His listing does not fulfill the requirements of ICZN Art. 74.7, and therefore the specimen is regarded as a syntype. The registration number quoted by him was incorrect. The type locality has not been found using modern gazetteers.

##### Current systematic position.

Odontostomidae, *Bahiensis longulus* (Pfeiffer, 1859).

#### Bulimus macgillivrayi

Pfeiffer, 1855

http://species-id.net/wiki/Bulimus_macgillivrayi

Figs 14B, 14v

Bulimus macgillivrayi Pfeiffer 1855b: 108, pl. 32 fig. 2.
Placostylus (Placocharis) macgillivrayi (Pfeiffer); Breure and Schouten 1985: 63 (lectotype designation).
Placocharis macgillivrayi (Pfeiffer); Delsaerdt 2010: 61, pl. 10 figs 9–11.

##### Type locality.

“Wanderer Bay, Guadalcanar, Salomon’s Islands (*Macgillivray*)”.

##### Label.

“Wanderer Bay, Guadal- / canar, Solomon’s Islds (Macgillivray)”. M.C. label style III.

##### Dimensions.

“Long. 59, diam. 22 mill.”; lectotype H 58.5, D 24.2, W 5.4.

##### Type material.

NHMUK 1975232, lectotype, and two paralectotypes, Macgillivray leg. (Cuming coll.).

##### Current systematic position.

Bothriembryontidae, *Placocharis macgillivrayi* (Pfeiffer, 1855).

#### Pupa spixii major

d’Orbigny, 1837

http://species-id.net/wiki/Pupa_spixii_major

Figs 22A–E, 22i

Helix spixii var. *major*d’Orbigny 1835: 21 [nomen nudum].
Pupa spixii var. α *major*d’Orbigny 1837 [1834–1847]: 320.
Pupa spixii (d’Orbigny); Gray 1854: 23 [partim].
Spixia striata (Spix); Breure 1976: 1158 [partim].

##### Type locality.

“...pays habité par les Guarayos, au sein des forêts humides des frontiers nord de la province de Chiquitos (république de Bolivia), et dans la province de Corrientes (république Argentine), en un bois voisin de la rivière de Santa-Lucia, au lieu dit “Pasto reito””; see Breure 1973: 122.

##### Label.

Two labels, “guarayos” and “corrientes, Rep. Argentina”, both in d’Orbigny’s handwriting.

##### Dimensions.

“Long. var. A, 35 millim., (...) Lat. var. A, 12 millim.”; lectotype H 34.8, D 11, W 10.6.

##### Type material.

NHMUK 1854.12.4.232, lectotype, matching best the original shell height; NHMUK 1854.12.4.232, six paralectotypes (d’Orbigny coll.).

##### Remarks.

In d’Orbigny (1835) only the name of this taxon is mentioned, together with a locality, thus it was invalidly published. The first valid publication is in d’Orbigny 1837 [1834–1847], where only a brief description was given but no figures of the ventral view of the shell (only a side view of a living snail). There is no explicit mentioning of “var. *major*” on the label, as d’Orbigny has done with varieties of other taxa; the only link are the localities quoted in his original paper. This taxon is here figured for the first time. As the lots referring to *Pupa spixii* refer to two taxa, a lectotype is here designated for each (design. n.); see also *minor* d’Orbigny.

##### Current systematic position.

Odontotomidae, *Spixia striata* (Spix, 1827).

#### Bulimus miltocheilus

Reeve, 1848

http://species-id.net/wiki/Bulimus_miltocheilus

Figs 10C, 10iii

Bulimus miltocheilus Reeve 1848 [1848–1850]: pl. 49 fig. 322; Breure and Schouten 1985: 70.
Aspastus miltocheilus (Reeve); Delsaerdt 2010: 21 (lectotype designation).

##### Type locality.

[Solomon Islands] “San Christoval, southeastern island of Solomon’s Group, northeast coast of New Holland”. Restricted to [Makira] “Kirakira (Wainoni-Bay)” by Rensch and Rensch (1935: 72).

##### Label.

“San Cristoval, Solomon Is.”. M.C. label style IV.

##### Dimensions.

Not given. Specimen figured herein H 68.3, D 25.0, W 6.0.

##### Type material.

NHMUK 20070072, two paralectotypes (Cuming coll.).

##### Remarks.

Delsaerdt (2010) has selected Reeve’s original figure as the lectotype.

##### Current systematic position.

Bothriembryontidae, *Aspastus miltocheilus* (Reeve, 1848).

#### Pupa spixii minor

d’Orbigny, 1837

http://species-id.net/wiki/Pupa_spixii_minor

Figs 21A–F, 21i

Helix spixii var. *minor*d’Orbigny 1835: 21 [nomen nudum].
Pupa spixii var. β *minor*d’Orbigny 1837 [1834–1847]: 320.
Pupa spixii (d’Orbigny); Gray 1854: 23 [partim].
Spixia striata (Spix); Breure 1976: 1158 [partim].

##### Type locality.

[Bolivia] “province de Chiquitos, entre Santo-Corazon et San-Juan”; see Breure 1973: 123.

##### Label.

“chiquitos, Bolivia”; in d’Orbigny’s handwriting.

##### Dimensions.

“Long. (...) var. B, 30 millim. Lat. (...) var. B, 7 millim.”; lectotype H 29.2, D 7.46, W 11.1.

##### Type material.

NHMUK 1854.12.4.231, lectotype and seven paralectotypes.

##### Remarks.

In d’Orbigny (1835) only the name of this taxon is mentioned, together with a locality, thus it was invalidly published. One specimen, which most closely matches the original shell height, is here designated as the lectotype. As the lots referring to *Pupa spixii* refer to two taxa, a lectotype is here designated for each (design. n.); see also *major* d’Orbigny. This taxon is here figured for the first time.

##### Current systematic position.

Odontostomidae, *Cyclodontina minor* (d’Orbigny, 1837).

#### Bulimus necouensis

Gassies, 1871

http://species-id.net/wiki/Bulimus_necouensis

Figs 8I, 8iv

Bulimus necouensis Gassies 1871: 191; Neubert et al. 2009: 68, fig. 75.

##### Type locality.

[New Caledonia] “Nécoué, Baie Lebris (Nouvelle-Calédonie)”.

##### Label. 

No locality information on the label, which gives the taxon name as “*Bulimus Nekouensis*”. A second label states “New Caledonia” but this is likely to have been added after the receipt of the material.

##### Dimensions.

“Long. 77 mill.; diam. maj. 33 mill.”; holotype H 73.9, D 33.6, W 6.5.

##### Type material.

NHMUK 1883.11.10.1175, holotype, E. Marie leg. (ex Gassies).

##### Remarks.

The specimen is marked “T” above the aperture on the last whorl. Neubert et al. (2009) have clarified the type status of this specimen and the taxonomic status of the taxon.

##### Current systematic position.

Bothriembryontidae, *Placostylus fibratus alexander* (Crosse, 1855).

#### Bulimus neglectus

Pfeiffer, 1847

http://species-id.net/wiki/Bulimus_neglectus

Figs 18H–I, 18i

Bulimus neglectus Pfeiffer 1847: 67.
Clessinia neglecta (Pfeiffer); Breure and Schouten 1985: 10 (lectotype designation); Simone 2006: 164, fig. 564.

##### Type locality.

[Brazil] “Brasilia”.

##### Label.

“Brazils”, taxon label in Pfeiffer’s handwriting. M.C. label style I.

##### Dimensions.

“Long 19, diam. 6 mill.”; lectotype H 16.8, D 5.18, W 8.2.

##### Type material.

NHMUK 1975263, lectotype and one paralectotype (Cuming coll.).

##### Remarks.

A note accompanies the material stating “types / var. designation not Pfeiffer’s”. However, the label on the back of the cardbox has Pfeiffer’s handwriting, and the type status of these specimens is not disputed here. Simone (2006) incorrectly gave the size of the lectotype as 21 mm height. This species is tentatively classified with *Bahiensis*, a group awaiting a generic level revision.

##### Current systematic position.

Odontostomidae, *Bahiensis neglecta* (Pfeiffer, 1847).

#### Buliminus nuptialis

Melvill and Ponsonby, 1894

http://species-id.net/wiki/Buliminus_nuptialis

Figs 6D–F, 6iii

Buliminus nuptialis Melvill and Ponsonby 1894: 92, pl. 1 fig. 5.
Prestonella nuptialis (Melvill and Ponsonby); Herbert 2007: 11, fig. 12–13.

##### Type locality.

[South Africa, Eastern Cape] “Craigie Burn, Somerset East (*Mrs. Mary Layard Barber*)”.

##### Label.

“Craigie Burn, Somerset / East, S. Africa”.

##### Dimensions.

“Long. 15, lat. 8.50 mill.”; holotype H 14.6, D 9.82, W 4.4.

##### Type material.

NHMUK 1905.1.26.29, holotype, Mrs. Mary Layard Barber (née Bowker) (ex E.L. Layard ex Ponsonby).

##### Remarks.

The data on the collector and the way this specimen has found its way to the NHMUK collection have been taken from Herbert (2007). The size of the specimen was erroneously given as “length 11.5 mm”.

##### Current systematic position.

Bothriembryontidae, *Prestonella nuptialis* (Melvill and Ponsonby, 1894).

#### Bulimus oblitus

Reeve, 1848

http://species-id.net/wiki/Bulimus_oblitus

Figs 18F–G, 18ii

Bulimus oblitus Reeve 1848 [1848–1850]: pl. 56 fig. 376.
Clessinia oblita (Reeve); Breure and Schouten 1985: 10 (lectotype designation); Simone 2006: 164, fig. 565.

##### Type locality.

“Brazil”.

##### Label.

“Brazil”. M.C. label style III.

##### Dimensions.

Not given. Lectotype H 20.9, D 6.27, W 9.2+.

##### Type material.

NHMUK 1975254, lectotype and two paralectotypes (Cuming coll.).

##### Remarks.

This species is tentatively classified with *Bahiensis*, awaiting a revision at generic level of this group. *Bulimus oblitus* is now considered a junior subjective synonym of *Bahiensis neglecta* (Pfeiffer, 1847) (synon.n.).

##### Current systematic position.

Odontostomidae, *Bahiensis neglecta* (Pfeiffer, 1847).

#### Bulimus occultus

Reeve, 1849

http://species-id.net/wiki/Bulimus_occultus

Figs 17C–D, 17ii

Bulimus occultus Reeve 1849 [1848–1850]: pl. 83 fig. 617.
Cyclodontina (Bahiensis) occultus (Reeve); Breure and Schouten 1985: 7, pl. 1 fig. 3 (lectotype designation).
Bahiensis occultus (Reeve); Simone 2006: 169, fig. 592.

##### Type locality.

“Brazil”.

##### Label.

“Brazil”. M.C. label style III.

##### Dimensions.

Not given. Lectotype H 23.6, D 5.8, W 9.0.

##### Type material.

NHMUK 1975258, lectotype (Cuming coll.).

##### Remarks.

See also the discussion under *Bulimus parallelus* Pfeiffer, 1857.

##### Current systematic position.

Odontostomidae, *Bahiensis occultus* (Reeve, 1849).

#### Bulimus ochrostoma

Garrett, 1872

http://species-id.net/wiki/Bulimus_ochrostoma

Figs 13B, 13iii

Bulimus ochrostoma Garrett 1872: 232, pl. 18 fig. 3.

##### Type locality.

[Fiji, Taviuni Island] “Tavinni [sic] Island, Viti Islands”.

##### Label.

“Taviuni, Viti”.

##### Dimensions.

“Length 29 mill., diam. 16 mill.”; specimen figured herein H 35.8, D 18.3, W 4.7.

##### Type material.

NHMUK 20030803, two probable syntypes (ex Taylor ex Garrett).

##### Remarks.

Garrett’s original description mentioned that types were in the author’s collection and in the ANSP. The specimens originate from Garrett and were probably part of the original series.

##### Current systematic position.

Bothriembryontidae, *Callistocharis ochrostoma* (Garrett, 1872).

#### Bulimus odontostoma

Sowerby I, 1824

http://species-id.net/wiki/Bulimus_odontostoma

Figs 24A–D, 24i

Bulimus odontostoma Sowerby I 1824: 59, pl. 5 fig. 3.

##### Type locality.

[Brazil] “Rio de Janeiro”.

##### Label.

“Brazil”, in a later hand. M.C. label style IV.

##### Dimensions.

“Axis 1 4/10, diam. 4/10 unc. [H 35.6, D 10.16 mm]”; specimen figured herein H 37.1, D 12.8, W 6.2.

##### Type material.

NHMUK 20100632, two possible syntypes (Cuming coll.).

##### Remarks.

According to Sowerby, he had seen “A few specimens...”. Since it is not unlikely that Sowerby made use of the Cuming collection to describe species (Petit 2009), the specimens are considered possible syntpes.

##### Current systematic position.

Odontostomidae, *Odontostomus gargantua* (Férussac, 1821).

#### Bulimus ouensis

Gassies, 1870

http://species-id.net/wiki/Bulimus_ouensis

Figs 7D, 7iv

Bulimus ouensis Gassies 1870: 142.
Placostylus (Placostylus) fibratus (Martyn); Breure and Schouten 1985: 60.
Placostylus fibratus fibratus (Martyn); Neubert et al. 2009: 50, fig. 10.

##### Type locality.

“Insula Ouen, Novae Caledoniae”.

##### Label.

Only a taxon label is present, with the name written in Gassies’ handwriting and marked “T”.

##### Dimensions.

“Long. 86, diam. maj. 41 mill.”; holotype H 83.7, D 40.7, W 7.3.

##### Type material.

NHMUK 1883.11.10.1176, holotype (ex Gassies).

##### Remarks.

The specimen is marked “T” on the last whorl beside the aperture. Gassies (1870) mentioned that he had seen only one specimen.

##### Current systematic position.

Bothriembryontidae, *Placostylus fibratus fibratus* (Martyn, 1784).

#### Bulimus parallelus

Pfeiffer, 1857

http://species-id.net/wiki/Bulimus_parallelus

Figs 17E–F, 17iii

Bulimus parallelus Pfeiffer 1857: 389; Breure and Schouten 1985: 7 (lectotype designation).
Moricandia parallela (Pfeiffer); Simone 2006: 172, fig. 606.

##### Type locality.

“St. Catherine’s, Brazil”.

##### Label.

“St. Catherines Brazil”, taxon label in Pfeiffer’s handwriting. M.C. label style III.

##### Dimensions.

“Long. 22, diam. 7 mill.”; lectotype H 22.5, D 7.17, W 7.3.

##### Type material.

NHMUK 1975256, lectotype and one paralectotype (Cuming coll.).

##### Remarks.

Simone (2006) has revalidated this taxon as a separate species, removing it from the synonymy of *Bahiensis occultus* (Reeve, 1849) (See also Pisbry 1901 [1901–1902]: 48), and placing it in the genus *Moricandia* Pilsbry & Vanatta, 1898. Pfeiffer’s type is somewhat stouter, but has the same characteristic brown blotch behind the lip. Tentatively, the synonymy with *Bulimus occultus* Reeve, 1849 is here retained.

##### Current systematic position.

Odontostomidae, *Bahiensis occultus* (Reeve, 1849).

#### Helix patagonica

d’Orbigny, 1835

http://species-id.net/wiki/Helix_patagonica

Figs 25E–G, 25ii

Helix patagonica d’Orbigny 1835: 22.
Bulimus patagonicus d’Orbigny 1837 [1834–1847]: 321, pl. 41 figs 17–18.
Plagiodontes patagonicus (d’Orbigny); Breure 1976: 1159, pl. 5 fig. 4, pl. 10 fig. 4 (lectotype designation).

##### Type locality.

[Argentina] “Patagonia”.

**Label.** “baie blanche [Bahia Blanca], Patagonia”, in d’Orbigny’s handwriting.

##### Dimensions.

“Longit. 22 1/2 millim., latit. 11 millim.”; specimen figured herein H 22.3, D 11.2, W 7.5.

##### Type material.

NHMUK 1854.12.4.235, five paralectotypes (d’Orbigny coll.).

##### Remarks.

The lectotype is in the MNHN collection (Breure 1976).

##### Current systematic position.

Odontostomidae, *Plagiodontes patagonica* (d’Orbigny, 1835).

#### Bulimus physoides

Reeve, 1849

http://species-id.net/wiki/Bulimus_physoides

Figs 2H–I, 2iv

Bulimus physoides Reeve 1849 [1848–1850]: pl. 70 fig. 507.
Bulimus (Liparus) physoides Reeve; E.A. Smith 1894: 95, pl. 7 fig. 30.

##### Type locality.

“—?”.

##### Label.

“W. Australia”, taxon label in Pfeiffer’s handwriting. M.C. label style III.

##### Dimensions.

Not given. Specimen figured herein H 19.2, D 11.22, W 5.0.

##### Type material.

NHMUK 1975224–1975225, three possible syntypes (Cuming coll.).

##### Remarks.

Reeve (legend to pl. 70: “Mus. Pfeiffer”) evidently based his description on material from Pfeiffer, who referred to “*physodes* (Menke mss.)”; this species was published as such by Menke (1843: 7), and Reeve’s name is therefore probably an emendation of Menke’s name. However, as Menke’s material is presumably lost (B.J. Smith 1992: 105), these specimens are interesting, since they may have originated via Pfeiffer from Menke’s collection.

##### Current systematic position.

Bothriembryontidae, *Bothriembryon melo* (Quoy and Gaimard, 1832).

#### Bulimus pinicola

Gassies, 1870

http://species-id.net/wiki/Bulimus_pinicola

Figs 8D, 8ii

Bulimus pinicola Gassies 1870: 142.
Placostylus (Placostylus) fibratus (Martyn); Breure and Schouten 1985: 60.
Placostylus fibratus guestieri (Gassies); Neubert et al. 2009: 55, fig. 21.

##### Type locality.

“Insula Pinorum, Novae Caledoniae”.

##### Label. 

Only a taxon label “Bulimus pinicula” in Gassies handwriting, marked “T”.

##### Dimensions.

“Long. 90, diam. maj. 44 mill.”; specimen figured herein H 92.8, D 44.3, W 6.7.

##### Type material.

NHMUK 1883.11.10.1164, one syntype (ex Gassies).

##### Remarks.

Marked “T” on last whorl besides aperture. Gassies (1870) stated that he had seen three specimens.

##### Current systematic position.

Bothriembryontidae, *Placostylus fibratus guestieri* (Gassies, 1869).

#### Placostylus ambagiosus priscus

Powell, 1938

http://species-id.net/wiki/Placostylus_ambagiosus_priscus

Fig. 12D

Placostylus ambagiosus priscus Powell 1938: 149, pl. 34 figs 9–10.

##### Type locality.

[New Zealand, North Island] “about three quarters of a mile east of the extreme north-western headland [Cape Maria van Diemen]”.

##### Label.

“Cape Maria van Diemen (mainland)”

##### Dimensions.

“Height, 77 mm.; breadth, 31 mm.”; specimen figured herein H 70.3, D 28.5, W 6.5.

##### Type material.

NHMUK 1939.3.17.18, one paratype (ex Powell).

##### Remarks. 

The holotype of this subspecies, which is supposedly of Pleistocene age, is in the Auckland Museum. Buckley et al. (2011) have revised the New Zealand *Placostylus* and showed that this taxon falls within the overall variation of *Placostylus ambagiosus* Suter, 1906.

##### Current systematic position.

Bothriembryontidae, *Placostylus ambagiosus* Suter, 1906.

#### Bulinus punctulifer

Sowerby I, 1833

http://species-id.net/wiki/Bulinus_punctulifer

Figs 5A–B, 5i

Bulinus punctulifer Sowerby I 1833 [1833–1838]: 36.
Plectostylus punctulifer (Sowerby); Breure 1979: 90.

##### Type locality.

“Chili, Questa Prado [Cuesta Lo Prado]”.

##### Label.

“Chili”. M.C. label style IV.

##### Dimensions.

“long. 1.5, lat. 0.75 poll. [H 38.0, D 19.0 mm]”; specimen figured herein H 46.5, D 21.7, W 5.3.

##### Type material.

NHMUK 1975171, eight syntypes (Cuming coll.).

##### Remarks.

The dimensions of the specimen figured herein have been taken from the largest specimen found in the lot.

##### Current systematic position.

Bothriembryontidae, *Plectostylus punctulifer* (Sowerby I, 1833).

#### Bulimus pyrostomus

Pfeiffer, 1860

http://species-id.net/wiki/Bulimus_pyrostomus

Figs 15C, 15iii

Bulimus pyrostomus Pfeiffer 1860: 137; Breure and Schouten 1985: 59, 72.
Placostylus (Santacharis) salomonis (Pfeiffer); Solem 1959: 132, pl. 8 fig. 5 (lectotype designation).
Santacharis salomonis (Pfeiffer); Delsaerdt 2010: 76, pl. 12 figs 13–14.

##### Type locality.

[Vanuatu, Erromanga] “Erumanga, New Hebrides”.

##### Label.

“Isle of Eurymanga, New Hebrides”, taxon label in Pfeiffer’s handwriting. M.C. label style III.

##### Dimensions.

“Long. 42, diam. 19 mill.”; lectotype H 41.5, D 22.0, W 5.3.

##### Type material.

NHMUK 1975235, lectotype and two paralectotypes (Cuming coll.).

##### Remarks.

According to Solem (1959) the “holotype of *pyrostomus* is more slender than most of the specimens seen, but it is not too atypical. The holotype of *salomonis* could not be located in the British Museum”. Solem based his comments on a photograph of the type and was possibly unaware of the existence of two additional specimens. His “holotype of *pyrostomus*” is here interpreted as a lectotype designation (Art. 74.6 ICZN), overruling the subsequent designation by Breure and Schouten (1985: 59). See also under *Bulimus salomonis*.

##### Current systematic position.

Bothriembryontidae, *Santacharis salomonis* (Pfeiffer, 1853).

#### Prestonella quadingensis

Connolly, 1929

http://species-id.net/wiki/Prestonella_quadingensis

Figs 6G–I, 6ii

**Figure 15.**
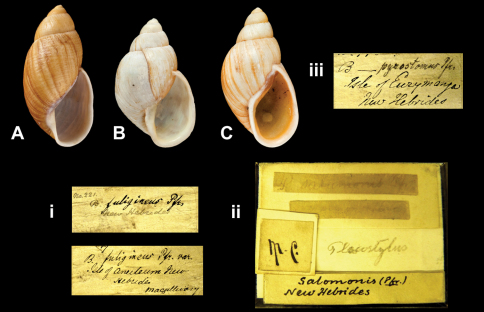
*Santacharis* species. **A, i** *Santacharis fuligineus* (Pfeiffer, 1853), lectotype NHM 1975226 (H = 39.4) **B–C** *Santacharis salomonis* (Pfeiffer, 1853) **B, ii** Lectotype NHM 1975234 (H = 33.6) **C, iii** Lectotype of *Bulimus pyrostomus* Pfeiffer, 1860 NHM 1975235 (H = 41.5).

**Figure 16.**
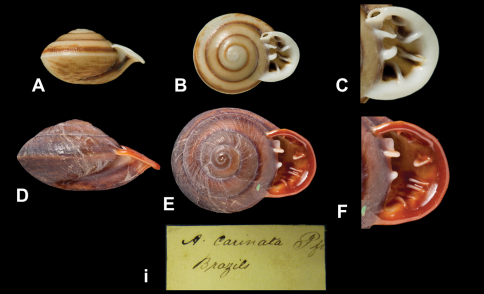
*Anostoma* species. **A–C, i** *Anostoma carinatum* (Pfeiffer, 1853), probable syntype NHM 20100509 (D = 24.3) **D–F** *Anostoma octodentatum brunneum* Verdcourt, 1991, holotype NHM 1990126 (D = 32.6).

**Figure 18.**
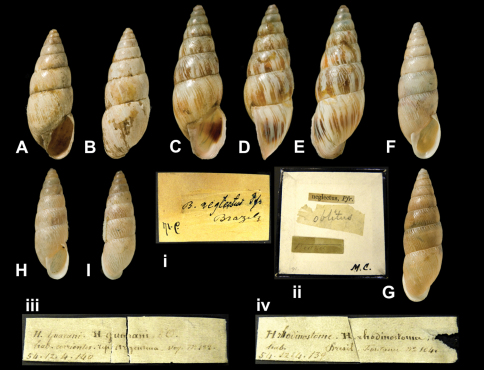
*Bahiensis* species. **A–B, iii** *Bahiensis guarani* (d’Orbigny, 1835), lectotype NHM 1854.12.4.140 (H = 20.5) **C–E, iv** *Bahiensis rhodinostoma* (d’Orbigny, 1835), lectotype NHM 1854.12.4.140 (H = 21.7) **F–I** *Bahiensis neglectus* (Pfeiffer, 1847) **F–G, ii** Lectotype of *Bulimus oblitus* Reeve, 1849 NHM 1975254 (H = 20.9) **H–I, i** Lectotype of *Bulimus neglectus* Pfeiffer, 1847 NHM 1975263 (H = 16.8).

Prestonella quadingensis Connolly 1929: 233, pl. 14 fig. 25; Herbert 2007: 13 figs 14–16.

##### Type locality.

[Lesotho, Quthing] “Basutoland, Quading”.

##### Label.

“Quading”.

##### Dimensions.

“Long. 12.3, lat. 7.3 mm”; specimen figured herein H 11.42, D 7.85, W 4.2.

##### Type material.

NHMUK 1937.12.30.7079–7080, two paratypes, E. Ford leg.

##### Remarks.

See Herbert (2007) for a discussion on the taxonomic status of this species.

##### Current systematic position.

Bothriembryontidae, *Prestonella quadingensis* Connolly, 1929.

#### Bulimus (Tomigerus) ramagei

E.A. Smith, 1890

http://species-id.net/wiki/Bulimus_ramagei

Figs 20A–B, 20i

Bulimus (Tomigerus) ramagei E.A. Smith 1890: 500, pl. 30 fig. 8.
Bonnanius ramagei (Smith); Simone 2006: 178, fig. 638.

##### Type locality.

[Brazil, Fernando Noronha] “Tobacco Point”.

##### Label.

“Fernando Noronha”.

##### Dimensions.

“Longit. 23 1/2 millim., diam. 16. / longit. 17 1/2 millim, diam. 12 1/2”; lectotype H 17.5, D 12.9, W 4.5.

##### Type material.

NHMUK 1888.6.27.163, lectotype; 1888.6.27.164–170, seven paralectotypes.

##### Remarks.

The specimen figured by E.A. Smith corresponds to the smallest specimen measured by him; this specimen is here selected lectotype (design.n.).

##### Current systematic position.

Odontostomidae, *Bonnanius ramagei* (E.A. Smith, 1890).

#### Succinea reflexa

Pfeiffer, 1842

http://species-id.net/wiki/Succinea_reflexa

Figs 5E–F, 5ii

Succinea reflexa Pfeiffer 1842: 56.
Plectostylus reflexus (Pfeiffer); Breure 1978: 202 (lectotype designation).

##### Type locality.

“Pichidanque prope Coquimbo, Chile”.

##### Label.

“Pichidanque near / Coquimbo, Chili”. M.C. label style IV.

##### Dimensions.

“Long. 39, diam. 17 mill.”; lectotype H 36.9, D 15.5, W 5.3.

##### Type material.

NHMUK 1975358, lectotype; 1975359, three paralectotypes (Cuming coll.).

##### Remarks.

The original label states “found on the leaves / of Pourretia Coarctata” [= *Puya chilensis* (Molina, 1782)]. This taxon is now considered a junior subjective synonym of *Bulinus broderipii* Sowerby I, 1832 (F. Cádiz, unpublished data).

##### Current systematic position.

Bothriembryontidae, *Plectostylus broderipii* (Sowerby I, 1832) (synon. n.).

#### Helix rhodinostoma

d’Orbigny, 1835

http://species-id.net/wiki/Helix_rhodinostoma

Figs 18C–E, 18iv

Helix rhodinostama [sic] d’Orbigny 1835: 20.
Bulimus rhodinostoma d’Orbigny 1837 [1834–1847]: 317, pl. 41 figs 6–8.
Bahiensis rhodinostomus (d’Orbigny); Simone 2006: 170, fig. 595.

##### Type locality.

[Brazil] “in imperio Brasiliano”.

##### Label.

“Brésil”, in d“Orbigny”s handwriting.

##### Dimensions.

“Longit. 21 millim., diam. 7 millim.”; lectotype H 21.7, D 7.25, W 8.1.

##### Type material.

NHMUK 1854.12.4.139, lectotype (d’Orbigny coll.).

##### Remarks.

The material consists of one specimen, corresponding to the figure in d’Orbigny 1837 [1834–1847]; it is here desingated lectotype (design.n.) as d’Orbigny did not state on how many specimens his description was based. Although d’Orbigny (1835) published this taxon as *Helix rhodinostama*, and this was not corrected by him in the same work, the prevailing usage of *rhodinostoma* makes this a justified emendation (Art. 33.2.3.1 ICZN).

##### Current systematic position.

Odontostomidae, *Bahiensis rhodinostoma* (d’Orbigny, 1835).

#### Bulimus rhodostomus

J.E. Gray, 1834

http://species-id.net/wiki/Bulimus_rhodostomus

Figs 3F–G, 3iv

Bulimus rhodostomus J.E. Gray 1834: 65; B.J. Smith 1992: 106.
Bothriembryon (Bothriembryon) rhodostomus (J.E. Gray); Breure 1978: 207 (lectotype designation); Breure 1979: 95.

##### Type locality.

[Australia] “Novâ Hollandiâ?”; see Remarks.

##### Label.

“Australia”, probably written by a later hand.

##### Dimensions.

“Axis 1 1/4, diam. 3/4 unc. [H 31.75, D 19.05 mm]”; lectotype H 31.1, D 18.0, W 5.4.

##### Type material.

NHMUK 1874.10.28.1, lectotype; 1975222, one paralectotype (ex Gray).

##### Remarks.

Iredale (1939: 19) has restricted the type locality to Western Australia, Goose Island.

##### Current systematic position.

Bothriembryontidae, *Bothriembryon rhodostomus* (J.E. Gray, 1834).

#### Bulimus (Bulimulus) ridleyi

E.A. Smith, 1890

http://species-id.net/wiki/Bulimus_ridleyi

Figs 20A–B, 20i

Bulimus (Bulimulus) ridleyi E.A. Smith 1890: 501, pl. 30 fig. 9.
Hyperaulax ridleyi (E.A. Smith); Simone 2006: 178, fig. 637.

##### Type locality.

[Brazil, Fernando Noronha] “on the north side of the island; also found at the base of the Peak, north side, (...) and on Rat Island”.

##### Label.

“Fernando Noronha”.

##### Dimensions.

“Longit. 12 millim., diam. 6”; lectotype H 11.46, D 5.9, W 5.3.

##### Type material.

NHMUK 1888.6.27.106, lectotype; 1888.6.27.107-110, four paralectotypes.

##### Remarks.

The specimen figured by E.A. Smith is here desingated lectotype (**design.n.**). The four paralectotypes are glued onto paper.

##### Current systematic position.

Odontostomidae, *Hyperaulax ridleyi* (E.A. Smith, 1890).

#### Partula salomonis

Pfeiffer, 1853

http://species-id.net/wiki/Partula_salomonis

Figs 15B, 15ii

Partula salomonis Pfeiffer 1853b: 446; Pfeiffer 1856, in Küster and Pfeiffer 1840–1865: 276, pl. 66 figs 10-11.
Placostylus (Santacharis) salomonis (Pfeiffer); Breure and Schouten 1985: 59 (lectotype designation).
Santacharis salomonis (Pfeiffer); Delsaerdt 2010: 76, pl. 12 figs 13-14.

##### Type locality.

“in insulis Salomonis” (see remarks).

##### Label.

“Solomon Ils” (badly faded). M.C. label style III.

##### Dimensions.

“Long. 34, diam. 15 mill.”; lectotype H 33.6, D 19.6, W 6.0.

##### Type material.

NHMUK 1975234, lectotype (Cuming coll.).

##### Remarks.

The specimen, which corresponds to the original measurements and which has been figured by Pfeiffer (1856), was designated lectotype by Breure and Schouten 1985. Dohrn (1862: 213) was the first to recognize the synonymy between *Partula salomonis* Pfeiffer, 1853 and *Bulimus pyrostomus* Pfeiffer, 1860, despite the erroneous locality given for the former taxon. The species only occurs on the New Hebrides (Solem 1959, Delsaerdt 2010).

##### Current systematic position.

Bothriembryontidae, *Santacharis salomonis* (Pfeiffer, 1853).

#### Bulimus sanchristovalensis

Cox, 1870

http://species-id.net/wiki/Bulimus_sanchristovalensis

Figs 12B, 12ii

Bulimus sanchristovalensis Cox 1870: 172, pl. 16 fig. 7.
Eumecostylus sanchristovalensis (Cox); Delsaerdt 2010: 37, pl. 4 figs 7–10 (lectotype designation).

##### Type locality.

“San Christoval, Solomon Islands”.

##### Label.

“Solomon Isls”, taxon label in Cox’ handwriting.

##### Dimensions.

“Diameter 1.20, length 1.60 (...) inch [H 40.6 D 30.5 mm; erroneous, see Delsaerdt 2010]”; lectotype H 67.7, D 27.6, W 5.4.

##### Type material.

NHMUK 1880.12.11.44, lectotype; 1880.12.11.45, one paralectotype, 20070069, five paralectotypes.

##### Remarks.

In addition, two further specimens are present; of which one is a probable paralectotype (1880.12.11.2) and the other (1877.7.23.23) is non-type material; however, as these specimens were previously mounted on the same board (possibly for display), but subsequently removed from the board, there is now no way of telling which specimen is which.

##### Current systematic position.

Bothriembryontidae, *Eumecostylus sanchristovalensis* (Cox, 1870).

#### Placostylus savesi

Crosse, 1886

http://species-id.net/wiki/Placostylus_savesi

Figs 9E, 9v

Placostylus savesi Crosse 1886: 163, pl. 7 figs 3–3a.
Placostylus (Placostylus) savesi Crosse; Breure and Schouten 1985: 63.
Placostylus eddystonensis savesi Crosse; Neubert et al. 2009: 96, fig. 152.

##### Type locality.

[New Caledonia] “in loco “Pouembou” dicto, Novae Caledoniae”.

##### Label.

“Pouembou (N. Cie)”, label in Crosse’s handwriting glued on dorsal side of shell.

##### Dimensions.

“Longit. 56 millim., diam. maj. 28.”; holotype H 57.5, D 30.0, W 6.0.

##### Type material.

NHMUK 1929.9.14.7, holotype (ex Crosse ex Marie).

##### Remarks.

The type status of this specimen has been discussed by Neubert et al. (2009); they concluded that it should be considered the holotype.

##### Current systematic position.

Bothriembryontidae, *Placostylus eddystonensis savesi* Crosse, 1886.

#### Bulimus sellersi

Cox, 1872

http://species-id.net/wiki/Bulimus_sellersi

Figs 10B, 10ii

Bulimus sellersi Cox 1872a: 644, pl. 52 fig. 3.
Aspastus sellersi (Cox); Delsaerdt 2010: 27, pl. 2 figs 15–19 (lectotype designation).

##### Type locality.

“Guadalcanar Island, Solomon Islands”.

##### Label.

“Solomon Isls”, taxon label “Placostylus / sellersi / Cox” (see remarks).

##### Dimensions.

“Length 1.90, breadth 0.66 of an inch [H 46.3, D 16.8 mm]”; lectotype H 44.4, D 18.9, W 5.5.

##### Type material.

NHMUK 20070067, lectotype; seven paralectotypes (ex Kennard ex Cox).

##### Remarks.

The specimens are accompanied by a second label “B. sellersi / Cox / Austr:”, which is possibly the original label of Cox. Furthermore, a third label. presumably made by Kennard, reads “Guadalcanar Cotypes Cox coll.”. The measurements of the lectotype as given by Delsaerdt (2010) appear to be erroneous (“The length of the lectotype is 50 mm”).

##### Current systematic position.

Bothriembryontidae, *Aspastus sellersi* (Cox, 1872).

#### Bulimus singularis

Morelet, 1857

http://species-id.net/wiki/Bulimus_singularis

Figs 9C, 9iii

Bulimus singularis Morelet 1857: 27; Breure and Schouten 1985: 72.
Placostylus porphyrostomus porphyrostomus (Pfeiffer); Neubert et al. 2009: 79, fig. 101.

##### Type locality.

[New Caledonia] “Portum Galliae, littore occidentali Nova-Caledoniae” [Nouméa].

##### Label.

“Nlle Calédonie” in Morelet`s handwriting.

##### Dimensions.

“Longit. 69, diam. 31 mill.”; holotype H 70.0 D 35.2, W 6.5.

##### Type material.

NHMUK 1893.2.4.1530, holotype (Morelet coll.).

##### Current systematic position.

Bothriembryontidae, *Placostylus porphyrostomus porphyrostomus* (Pfeiffer, 1851).

#### Bulimus ouveanus sinistrorsa

Crosse, 1884

http://species-id.net/wiki/Bulimus_ouveanus_sinistrorsa

Figs 8E–F

Bulimus ouveanus sinistrorsa Crosse 1884: 328, pl. 7 figs 3–3a.
Placostylus fibratus ouveanus (Dotzauer in Mousson); Neubert et al. 2009: 74, fig. 97.

##### Type locality.

[New Caledonia] “Lifou, une des îles Loyalty”.

##### Label.

No locality; taxon label in Crosse’s handwriting.

##### Dimensions.

“Longueur totale de la coquille 47 millimètres, plus grand diamètre 23”; specimen figured herein H 49.2, D 26.0, W 6.2.

##### Type material.

NHMUK 1929.9.14.8, one specimen, E. Marie leg. (Crosse coll.).

##### Remarks.

Crosse described this specimen as monstruosity. Neubert et al. (2009) correctly stated that this taxon name was proposed for a teratological specimen and is thus unavailable under ICZN Art. 1.3.2.

##### Current systematic position.

Bothriembryontidae, *Placostylus fibratus ouveanus* (Dotzauer in Mousson, 1869).

#### Bulimus souvillei

Morelet, 1857

http://species-id.net/wiki/Bulimus_souvillei

Figs 9B, 9ii

Bulimus souvillei Morelet 1857: 26.
Placostylus (Placostylus) souvillei (Morelet); Breure and Schouten 1985: 63 (lectotype designation).
Placostylus fibratus souvillei (Morelet); Neubert et al. 2009: 63, figs 55–68.

##### Type locality.

[New Caledonia] “ad Sanctam-Mariam de Balade, Novae-Caledoniae ore occidentale” [see remarks].

##### Label.

“Ste Marie de Balade. Nlle Calédonie”, in Morelet`s handwriting.

##### Dimensions.

“Longit. 118; diam. maj. 62 mill.”; lectotype H 116.5, D 66.5, W 6.8.

##### Type material.

NHMUK 1893.2.4.1529, lectotype (ex Morelet).

##### Remarks.

The type locality is erroneous according to Neubert et al. (2009), “as *f. souvillei* does not live north of the Mt. Humboldt area”.

##### Current systematic position.

Bothriembryontidae, *Placostylus fibratus souvillei* (Morelet, 1857).

#### Liparus spenceri

Tate, 1894

http://species-id.net/wiki/Liparus_spenceri

Figs 2J–K, 2v

Liparus spenceri Tate 1894: 192; Tate 1896: 202, pl. 17 fig. 3; B.J. Smith 1992: 107.

##### Type locality.

[Northern Territory, near Alice Springs] “Glen of Palms by the junction with Palm Creek” (Tate 1896: 202).

##### Label. 

“Glen of Palms”, presumably in Tate’s handwriting.

**Dimensions.** “Length, 20; width 12.5 (...) mm”; specimen figured herein H 17.5, D 12.2, W 4.4.

##### Type material.

NHMUK 1895.10.1.202–207, six paratypes (ex W.A. Horn).

##### Remarks.

This material was collected during the Horn Scientific Expedition to Central Australia (1894). Three of the specimens are subadult or juvenile. Further type material is present in the South Australia Museum in Adelaide (Breure and Whisson in press).

##### Current systematic position.

Bothriembryontidae, *Bothriembryon spenceri* (Tate, 1894).

#### Bulimus strangei

Pfeiffer, 1855

http://species-id.net/wiki/Bulimus_strangei

Figs 14D, 14iv

Bulimus strangei Pfeiffer 1855a: 8.
Placocharis strangei (Pfeiffer); Delsaerdt 2010: 73, pl. 12 figs 5–9 (lectotype designation).

##### Type locality.

[Solomon Islands, Simbo] “Eddystone Island, Australian Seas”.

##### Label.

“Eddystone Isld / Australian Seas”. M.C. label style III.

##### Dimensions.

“Long. 46, diam. 17 mill.”; lectotype H 45.9, D 20.7, W 5.4.

##### Type material.

NHMUK 20100652, lectotype and two paralectotypes (Cuming coll.).

##### Remarks.

Delsaerdt (2010) selected Pfeiffer’s figure (1856 in Küster and Pfeiffer 1840–1865: pl. 16 figs 11–12) as lectotype. He remarked that the whereabouts of the type are unknown. Three specimens were found in the NHM Mollusca General Collection, which originate from Cuming’s collection and have the label in agreement with Pfeiffer’s type locality. One of the specimens corresponds to the figure in Delsaerdt representing the lectotype; its measurements fit with Pfeiffer’s and it is now considered the lectotype despite the fact that the taxon label is not in Pfeiffer’s handwriting.

##### Current systematic position.

Bothriembryontidae, *Placocharis strangei* (Pfeiffer, 1855).

#### Placostylus (Callistocharis) subroseus

Fulton, 1915

http://species-id.net/wiki/Placostylus_subroseus

Figs 13C, 13ii

Placostylus (Callistocharis) subroseus Fulton 1915: 242, fig; Breure and Schouten 1985: 73.

##### Type locality.

[Fiji] “Viti Islands”.

##### Label.

“Viti Ids”.

##### Dimensions.

“Alt. 44, diam. maj. 20 mm.”; holotype H 43.8, D 20.9, W 5.2.

##### Type material.

NHMUK 1919.12.31.50, holotype (ex Fulton).

##### Current systematic position.

Bothriembryontidae, *Callistocharis subroseus* (Fulton, 1915).

#### Bulimus superfasciatus

Gassies, 1871

http://species-id.net/wiki/Bulimus_superfasciatus

Figs 7C, 7iii

Bulimus superfasciatus Gassies 1871: 190; Breure and Schouten 1985: 60.
Placostylus fibratus fibratus (Martyn); Neubert et al. 2009: 50, fig. 16

##### Type locality.

[New Caledonia] “Boulari”.

##### Label.

No locality given. Taxon label in Gassies’ hand, and marked with “T”.

##### Dimensions.

“Long. 80 mill.; diam. maj. 37 mill.”; specimen figured herein H 77.1, D 39.8, W 5.8.

##### Type material.

NHMUK 1883.11.10.1165, syntype (ex Gassies).

##### Remarks.

The characteristic “T” mark confirms the type status of this specimen.

##### Current systematic position.

Bothriembryontidae, *Placostylus fibratus fibratus* (Martyn, 1784).

#### Bulimus tasmanicus

Pfeiffer, 1853

http://species-id.net/wiki/Bulimus_tasmanicus

Figs 3H–I, 3iii

Bulimus tasmanicus Pfeiffer 1853c: 260; B.J. Smith 1992: 108.

##### Type locality.

[Australia, Tasmania] “Van Diemen’s Land” (see remarks).

##### Label.

“Van Diemens / isl.”, taxon label in Pfeiffer’s handwriting. M.C. label style III.

##### Dimensions.

“Long. 25, diam. 11 mill.”; specimen figured herein H 29.3, D 15.1, W 5.4.

##### Type material.

NHMUK 1981204, one syntype (Cuming coll.).

##### Remarks.

The material (two specimens) is accompanied by two labels in Pfeiffer’s handwriting. One states “B. Tasmanicus / Pfr. Van Diemens / isl.”; this is considered the original label as it is entirely written in Pfeiffer’s hand and the specimen is considered to be a syntype. The second label reads “B. Tasmanicus Pfr. var. / Isle of Ianna / New Caledonia”; only the taxon name (“Tasmanicus Pfr. var.”) is in Pfeiffer’s handwriting and this material is not considered to be a syntype as the locality seems to be erroneous.

Kershaw (1987) has restricted the type locality to Maria Island.

##### Current systematic position.

Bothriembryontidae, *Bothriembryon tasmanicus* (Pfeiffer, 1853).

#### Odontostomus (Moricandia) toleratus

Fulton, 1903

http://species-id.net/wiki/Odontostomus_toleratus

Figs 23C–D, 23ii

Odontostomus (Moricandia) toleratus Fulton 1903: 100, pl. 9 fig. 2.
Moricandia tolerata (Fulton); Simone 2006: 172, fig. 607.

##### Type locality.

“Brazil”.

##### Label.

“Brazil”, in Fulton’s handwrting.

##### Dimensions.

“Maj. diam. 9; alt. 32 millim.”; holotype H 31.9, D 9.54, W 8.8.

##### Type material.

NHMUK 1903.11.20.34, holotype (ex Fulton).

##### Remarks.

The label indicates “Type”.

##### Current systematic position.

Odontostomidae, *Moricandia tolerata* (Fulton, 1903).

#### Tomogeres turbinatus

Pfeiffer, 1845

http://species-id.net/wiki/Tomogeres_turbinatus

Figs 19F, 19ii

Tomogeres turbinatus Pfeiffer 1845a: 45; Pfeiffer in Philippi 1846 [1845–1847]: 131, pl. 8 fig. 13.
Tomigerus (Tomigerus) turbinatus (Pfeiffer); Breure and Schouten 1985: 24, pl. 1 figs 8–10 (lectotype designation).
Biotocus turbinatus (Pfeiffer); Simone 2006: 178, fig. 634.

##### Type locality.

“In Brasiliâ”.

##### Label.

“Brazils”. M.C. label style IV.

##### Dimensions.

“Diam. maj. 11 (...) alt. 10 mill.”; lectotype H 9.68, D 12.56, W 5.0.

##### Type material.

NHMUK 1973107, lectotype and two paralectotypes (Cuming coll.).

##### Remarks.

The material is not accompanied by a label in Pfeiffer’s handwriting. The lectotype corresponds to the figure given by Pfeiffer in Philippi 1846 [1845–1847].

##### Current systematic position.

Odontostomidae, *Biotocus turbinatus* (Pfeiffer, 1845).

#### Bulimus turneri

Pfeiffer, 1860

http://species-id.net/wiki/Bulimus_turneri

Figs 13D, 13v

Bulimus turneri Pfeiffer 1860: 138, pl. 51 fig. 10; Breure and Schouten 1985: 73.
Placostylus (Poecilocharis) turneri (Pfeiffer); Solem 1959: 137, pl. 8 figs 1–2.

##### Type locality.

[Vanuatu, Erromanga] “Erumanga, New Hebrides”.

##### Label.

“Euramanga, New Hebr”. M.C. label style III.

##### Dimensions.

“Long. 32, diam. 17 mill.”; lectotype H 32.9, D 19.2, W 3.9.

##### Type material.

NHMUK 1975237, lectotype and two paralectotypes, Turner leg. (Cuming coll.).

##### Remarks.

The holotype designation (Solem 1959) has to be interpreted as lectotype designation (Art. 74.6 ICZN). Of the paralectotypes, one is subadult, the other juvenile.

##### Current systematic position.

Bothriembryontidae, *Poecilocharis turneri* (Pfeiffer, 1860).

#### Succinea variegata

Pfeiffer, 1842

http://species-id.net/wiki/Succinea_variegata

Figs 5C–D, 5iii

Succinea variegata Pfeiffer 1842: 56; Pfeiffer 1843: 187.
Plectostylus variegatus (Pfeiffer); Breure 1978: 202, pl. 9 figs 17–18 (lectotype designation).

##### Type locality.

“Chile prov. septembr. (*Bridges*)”.

##### Label.

“the valleys north of Coquimbo / found in the crevices of roc[ks]”. M.C. label style IV.

##### Dimensions.

“Long. 48, diam. 23 mill.”; lectotype H 46.0, D 24.8, W 5.3.

##### Type material.

NHMUK 1975362, lectotype; 1975363, three paralectotypes (Cuming coll.).

##### Remarks.

Pfeiffer’s description was read before the Zoological Society of London on 13 December 1842, but appeared in print in 1843. In this paper he gives the exact text as shown on the label. The locality label does not seem to have the taxon name in Pfeiffer’s hand; however, there is a second label in his handwriting “B. variegatus / Pfr.”. Therefore we do not question the type status of these specimens. This taxon is now considered a junior subjective synonym of *Bulinus broderipii* Sowerby I, 1832 (F. Cádiz, unpublished data).

##### Current systematic position.

Bothriembryontidae, *Plectostylus broderipii* (Sowerby I, 1832) (synon. n.).

## Supplementary Material

XML Treatment for Bulimus ouveanus alba

XML Treatment for Bulimus angasianus

XML Treatment for Bulimus asopeus

XML Treatment for Eumecostylus vicinus backhuysi

XML Treatment for Bulimus bairdii

XML Treatment for Bulimus (Mesembrinus?) bowkeri

XML Treatment for Bulimus (Liparus) brazieri

XML Treatment for Bulinus broderipii

XML Treatment for Anostoma octodentatum brunneum

XML Treatment for Bulimus bulbulus

XML Treatment for Placostylus calus

XML Treatment for Anostoma carinatum

XML Treatment for Bulimus catharinae

XML Treatment for Bulimus charpentieri

XML Treatment for Cyclodontina (Spixia) corderoi

XML Treatment for Bulimus corpulentus

XML Treatment for Bulimus costatus

XML Treatment for Bulinus coturnix

XML Treatment for Bulimus crassilabrum

XML Treatment for Tomigerus cumingii

XML Treatment for Bulimus cuninculinsulae

XML Treatment for Placostylus (Euplacostylus) cylindricus

XML Treatment for Pupa dentata

XML Treatment for Bulimus dux

XML Treatment for Bulimus eddystonensis

XML Treatment for Bulimus edwardsianus

XML Treatment for Succinea elegans

XML Treatment for Bulimus broderipi elongata

XML Treatment for Bulimus falcicula

XML Treatment for Placostylus (Eumecostylus) foxi

XML Treatment for Bulimus fuligineus

XML Treatment for Bulimus fuscagula

XML Treatment for Bulimus souvillei gatopensis

XML Treatment for Bulimus fibratus grammica

XML Treatment for Bulimus grayanus

XML Treatment for Helix guarani

XML Treatment for Placostylus guppyi

XML Treatment for Bulimus hargravesi

XML Treatment for Bulimus infundibulum

XML Treatment for Bulimus kingii

XML Treatment for Bulimus koroensis

XML Treatment for Bulimus (Charis) kreftii

XML Treatment for Bulimus leeuwinensis

XML Treatment for Bulimus longulus

XML Treatment for Bulimus macgillivrayi

XML Treatment for Pupa spixii major

XML Treatment for Bulimus miltocheilus

XML Treatment for Pupa spixii minor

XML Treatment for Bulimus necouensis

XML Treatment for Bulimus neglectus

XML Treatment for Buliminus nuptialis

XML Treatment for Bulimus oblitus

XML Treatment for Bulimus occultus

XML Treatment for Bulimus ochrostoma

XML Treatment for Bulimus odontostoma

XML Treatment for Bulimus ouensis

XML Treatment for Bulimus parallelus

XML Treatment for Helix patagonica

XML Treatment for Bulimus physoides

XML Treatment for Bulimus pinicola

XML Treatment for Placostylus ambagiosus priscus

XML Treatment for Bulinus punctulifer

XML Treatment for Bulimus pyrostomus

XML Treatment for Prestonella quadingensis

XML Treatment for Bulimus (Tomigerus) ramagei

XML Treatment for Succinea reflexa

XML Treatment for Helix rhodinostoma

XML Treatment for Bulimus rhodostomus

XML Treatment for Bulimus (Bulimulus) ridleyi

XML Treatment for Partula salomonis

XML Treatment for Bulimus sanchristovalensis

XML Treatment for Placostylus savesi

XML Treatment for Bulimus sellersi

XML Treatment for Bulimus singularis

XML Treatment for Bulimus ouveanus sinistrorsa

XML Treatment for Bulimus souvillei

XML Treatment for Liparus spenceri

XML Treatment for Bulimus strangei

XML Treatment for Placostylus (Callistocharis) subroseus

XML Treatment for Bulimus superfasciatus

XML Treatment for Bulimus tasmanicus

XML Treatment for Odontostomus (Moricandia) toleratus

XML Treatment for Tomogeres turbinatus

XML Treatment for Bulimus turneri

XML Treatment for Succinea variegata

## Figures and Tables

**Figure 2.**
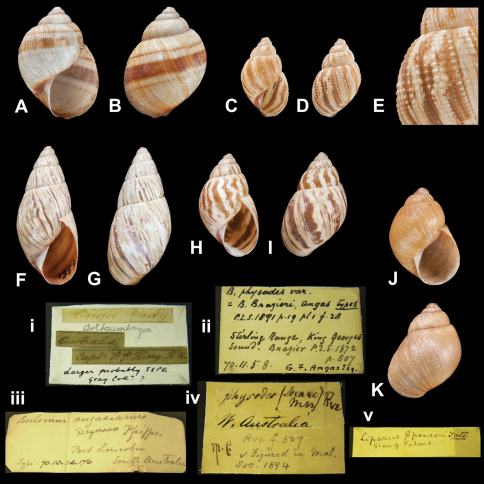
*Bothriembryon* species. **A–B, iii** *Bothriembryon angasianus* (Pfeiffer, 1864), syntype NHM 1870.10.26.276 (H = 20.6) **C–E, ii** *Bothriembryon brazieri* (Angas, 1871, lectotype NHM 1870.11.5.8 (H = 14.3) **E** detail of sculpture (× 3.5) **F–G, i** *Bothriembryon kingii* (J.E. Gray, 1825), lectotype NHM 1975910 (H = 19.1) **H–I, iv** *Bothriembryon melo* (Quoy and Gaimard, 1832), lectotype of *Bulimus physoides* Reeve, 1848 NHM 1975224 (H = 19.2) **J–K, v** *Bulimus spenceri* (Tate, 1894), syntype NHM 1895.10.2.202 (H = 17.5).

**Figure 3.**
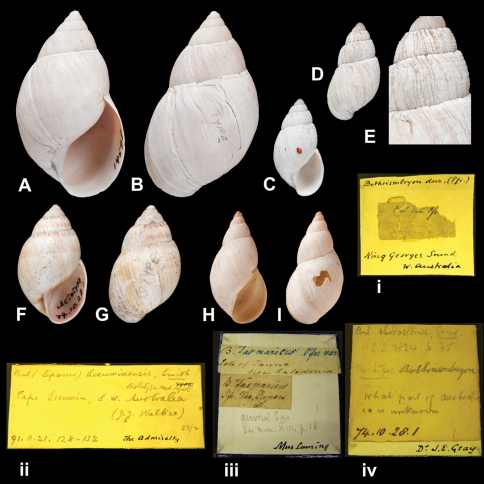
*Bothriembryon* species. **A–B, i** *Bothriembryon dux* (Pfeiffer, 1861), lectotype NHM 1975598 (H = 48.5) **C–E, ii** *Bothriembryon leeuwinensis* (E.A. Smith, 1894), lectotype NHM 1881.11.21.128 (H = 27.0); **E** detail of sculpture (× 6) **F–G, iv** *Bothriembryon rhodostomus* (J.E. Gray, 1834), lectotype NHM 1874.10.28.1 (H = 31.1) **H–I, iii** *Bothriembryon tasmanicus* (Pfeiffer, 1853), syntype NHM 1981204 (H = 30.1).

**Figure 4.**
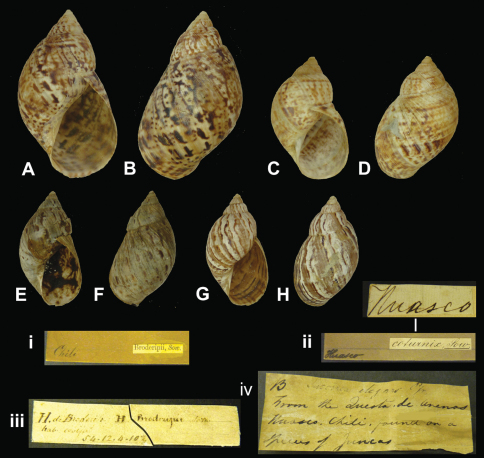
*Plectostylus* species. **A–B, i** *Plectostylus broderipii* (Sowerby I, 1832), lectotype NHM 20100655 (H = 44.5) **C–D, ii** *Plectostylus coturnix* (Sowerby, 1832), possible syntype NHM 20100620 (H = 32.4) **E–H** *Plectostylus broderipii* (Sowerby I, 1832) **E–F, iii** Lectotype of *Bulimus broderipi* var. *elongata* d’Orbigny, 1837 NHM 1854.12.4.102 (H = 30.5) **G–H, iv** Lectotype of *Succinea elegans* Pfeiffer, 1842 NHM 1975360 (H = 30.0).

**Figure 5.**
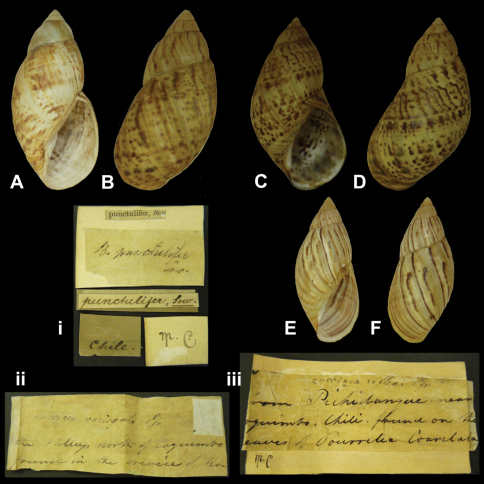
*Plectostylus* species. **A–B, i** *Plectostylus punctulifer* (Sowerby I, 1833), syntype NHM 1975171 (H = 46.5) **C–F** *Plectostylus broderipii* (Sowerby I, 1832) **C–D, iii** Lectotype of *Bulimus variegatus* Pfeiffer, 1842 NHM 1975362 (H = 46.0) **E–F, ii** Lectotype of *Bulimus reflexus* Pfeiffer, 1842 NHM 12975358 (H = 36.9).

**Figure 6.**
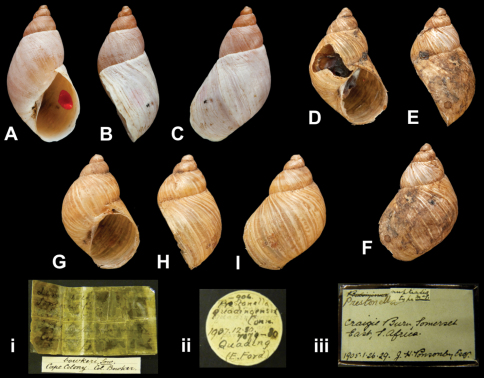
*Prestonella* species. **A–C, i** *Prestonella bowkeri* (Sowerby, 1890), lectotype NHM 1889.11.4.9 (H = 19.6) **D–F, iii** *Prestonella nuptialis* (Melvill and Ponsonby, 1894), holotype NHM 1905.1.26.29 (H = 14.6) **G–I, ii** *Prestonella quadingensis* Connolly, 1929, paratype NHM 1937.12.30.7079 (H = 11.4).

**Figure 7.**
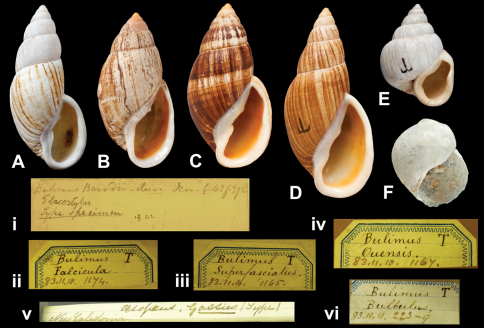
*Placostylus fibratus fibratus* (Martyn, 1784). **A, i** *Bulimus bairdii* Reeve, 1848, lectotype NHM 1975223 (H = 78.1) **B, ii** *Bulimus falcicula* Gassies, 1871, syntype NHM 1883.11.10. (H = 74.5) **C, iii** *Bulimus superfasciatus* Gassies, 1871, syntype NHM 1883.11.10.1167 (H = 77.1) **D, iv** *Bulimus ouensis* Gassies, 1870, holotype NHM 1883.11.10.1176 (H = 83.7) **E, v** *Bulimus asopeus* Gassies, 1871, holotype NHM 1883.11.10.1173 (H = 49.7) **F, vi** *Bulimus bulbulus* Gassies, 1871, syntype NHM 1883.11.10.223 (H = 4.13). All reduced in size.

**Figure 9.**
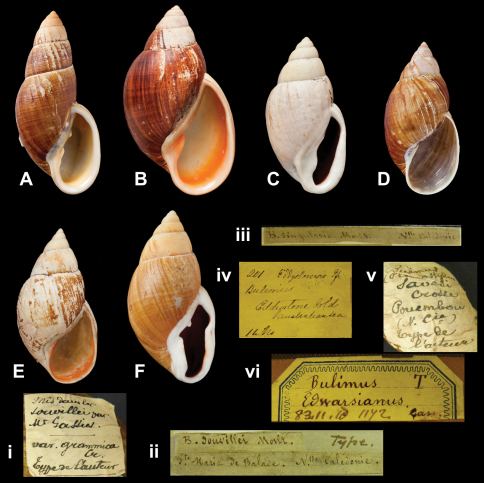
*Placostylus* species. **A, i** *Placostylus fibratus goroensis* (Souverbie in Souverbie and Montrouzier, 1870), syntype of *Bulimus fibratus grammica* Crosse, 1870 NHM 1929.9.14.15 (H = 111.3) **B, ii** *Placostylus fibratus souvillei* (Morelet, 1857), lectotype NHM 1893.2.4.1529 (H = 116.5) **C, iii** *Placostylus porphyrostomus porphyrostomus* (Pfeiffer, 1851), holotype of *Bulimus singularis* Morelet, 1857 NHM 1893.2.4.1530 (H = 70.0) **D, iv** *Placostylus eddystonensis eddystonensis* (Pfeiffer, 1855), lectotype NHM 1975230 (H = 73.3) **E, v** *Placostylus eddystonensis savesi* (Crosse, 1886), holotype NHM 1929.9.14.7 (H = 57.5) **F, vi** *Placostylus caledonicus* (Petit de la Saussaye, 1845), possible syntype of *Bulimus edwardsianus* Gassies, 1863 NHM 1883.11.10.1172 (H = 72.0). All reduced in size.

**Figure 10.**
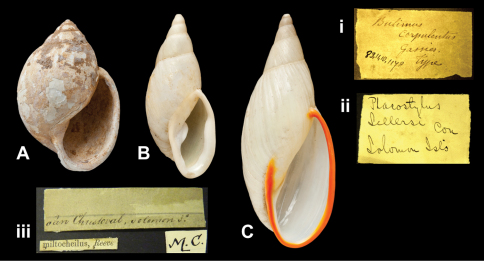
*Placostylus* and *Aspastus* species. **A, i** *Placostylus corpulentus* (Gassies, 1871), syntype NHM 1883.11.10.1173 (H = 94.0). Reduced in size **B, ii** *Aspastus sellersi* (Cox, 1871), lectotype NHM 2007069 (H = 44.4) **C, iii** *Aspastus miltocheilus* (Reeve, 1848), lectotype NHM 2007072 (H = 68.3).

**Figure 11.**
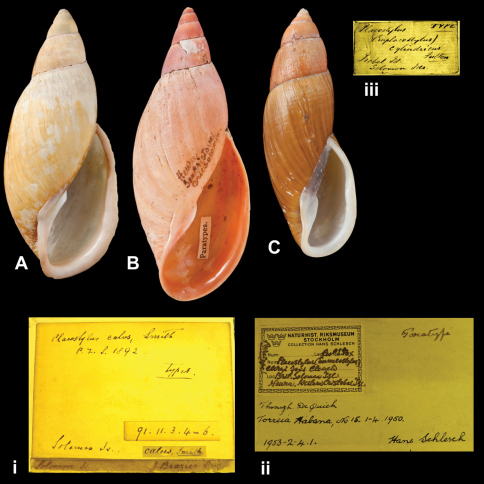
*Eumecostylus* species. **A, i** *Eumecostylus calus* (E.A. Smith, 1891), lectotype NHM 1891.11.3.4 (H = 78.0) **B, ii** *Eumecostylus foxi* (Clench, 1941), paratype NHM 1953.2.4.1 (H = 82.3) **C, iii** *Eumecostylus cylindricus* (Fulton, 1907), lectotype NHM 1907.5.3.160 (H = 71.3).

**Figure 12.**
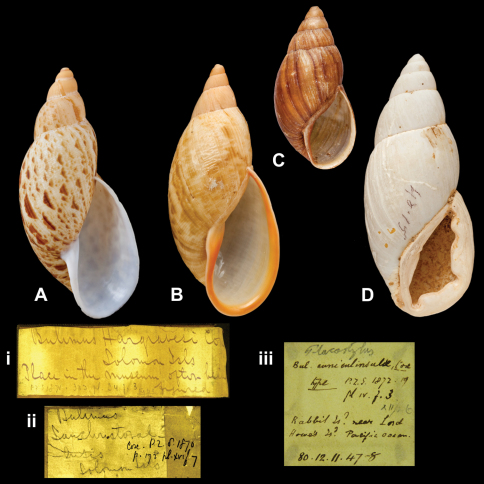
*Eumecostylus* and *Placostylus* species. **A, i** *Eumecostylus hargravesi* (Cox, 1871), lectotype NHM 1880.12.11.43 (H = 65.0) **B, ii** *Eumecostylus sanchristovalensis* (Cox, 1870), lectotype NHM 1880.12.11.44 (H = 67.7) **C, iii** *Placostylus cuninculinsulae* (Cox, 1872), lectotype NHM 1880.12.11.47 (H = 41.6) **D** *Placostylus ambagiosus* Suter, 1906, paratype of *Placostylus ambagiosus priscus* Powell, 1938, NHM 1939.3.17.18 (H = 70.3).

**Figure 13.**
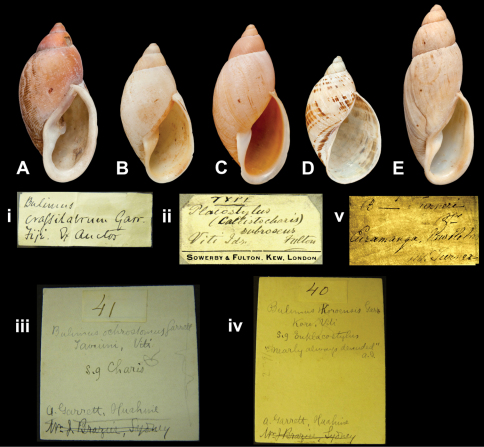
**A–C** *Callistocharis* species **A, i** *Callistocharis crassilabrum* (Garrett, 1872), syntype NHM 20030801 (H = 44.0) **B, iii** *Callistocharis ochrostoma* (Garrett, 1872), probable syntype NHM 20030803 (H = 35.8) **C, ii** *Callistocharis subroseus* (Fulton, 1915), holotype NHM 1919.12.31.50 (H = 43.8) **D, v** *Poecilocharis turneri* (Pfeiffer, 1860), lectotype NHM 1975237 (H = 32.9) **E, iv** *Euplacostylus koroensis* (Garrett, 1872), probable syntype NHM 20030802 (H = 51.2).

**Figure 17.**
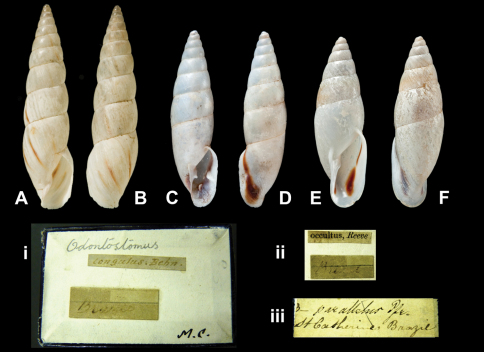
*Bahiensis* species. **A–B, i** *Bahiensis longulus* (Pfeiffer, 1859), syntype NHM 1975252 (H = 29.8) **C–F** *Bahiensis occultus* (Reeve, 1849) **C–D, ii** Lectotype of *Bulimus occultus* Reeve, 1849 NHM 1975258 (H = 23.6) **E–F, iii** Lectotype of *Bulimus parallelus* Pfeiffer, 1857 (H = 22.5).

**Figure 19.**
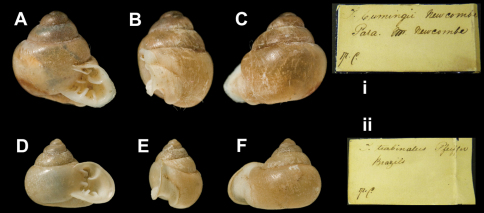
*Biotocus* species. **A–C, i** *Biotocus cumingii* (Pfeiffer, 1849), syntype NHM 1975787 (H = ). **D–F, ii** *Biotocus turbinatus* (Pfeiffer, 1845), lectotype NHM 1975253 (H = 9.68).

**Figure 20.**
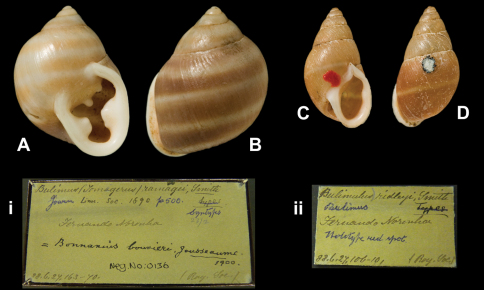
**A–B, i** *Bonnanius ramagei* (E.A. Smith, 1890), lectotype NHM 1888.6.27.163 (H = 17.5) **C–D, ii** *Hyperaulax ridleyi* (E.A. Smith, 1890, lectotype NHM 1888.6.27.106 (H = 11.46).

**Figure 21.**
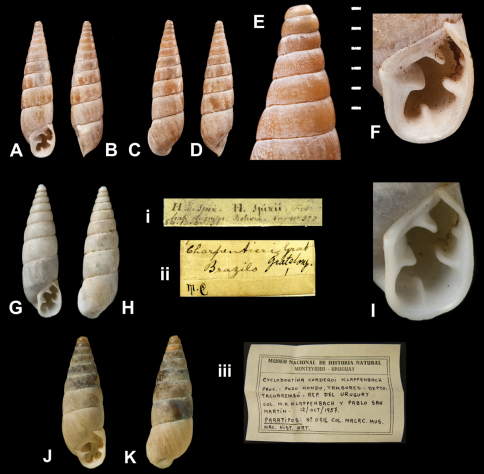
*Cyclodontina* species. **A–F, i** *Cyclodontina minor* (d’Orbigny, 1837), lectotype NHM 1854.12.4.231 (H = 29.2) **E** apical whorls (scale equals 5 mm) **F** detail of aperture **G–I, ii** *Cyclodontina charpentieri* (Pfeiffer, 1850), possible syntype NHM 1975585 (H = 17.9) **I** detail of aperture **J–K, iii** *Cyclodontina corderoi* Klappenbach, 1958, paratype NHM 1961679 (H = 16.1).

**Figure 22.**
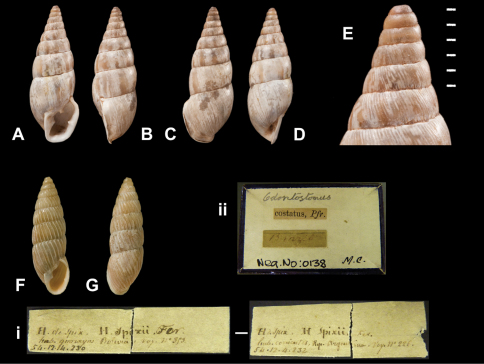
**A–E, i** *Spixia striata* (Spix, 1827), lectotype of *Pupa spixii major* (d’Orbigny, 1837) NHM 1854.12.4.232 (H = 34.8) **E** apical whorls (scale equals 5 mm) **F–G, ii** *Clessinia costata* (Pfeiffer, 1848), lectotype NHM 1975250 (H = 17.8).

**Figure 23.**
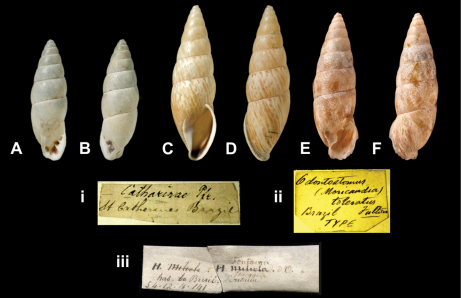
**A–B, i** *Cyclodontina fusiformis* (Menke, 1828), lectotype of *Bulimus catharinae* Pfeiffer, 1857 NHM 1975261 (H = 24.4) **C–D, ii** *Moricandia tolerata* (Fulton, 1903), holotype NHM 1903.11.20.34 (H = 31.9) **E–F, iii** *Bahiensis fuscagula* (d’Orbigny, 1837), lectotype NHM 1854.12.4.141 (H = 30).

**Figure 24.**
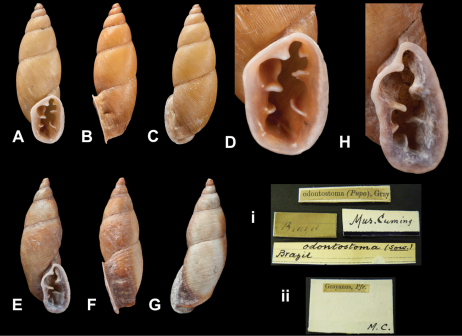
*Odontostomus* species. **A–D, i** *Odontostomus gargantua* (Férussac, 1821), possible syntype of *Bulimus odontostomus* Sowerby I, 1824 NHM 20100632 (H = 37.1) **D** detail of aperture **E–H, ii** *Odontostomus grayanus* (Pfeiffer, 1845), syntype NHM 20110387 (H = 34.1) **H** detail of aperture.

**Figure 25.**
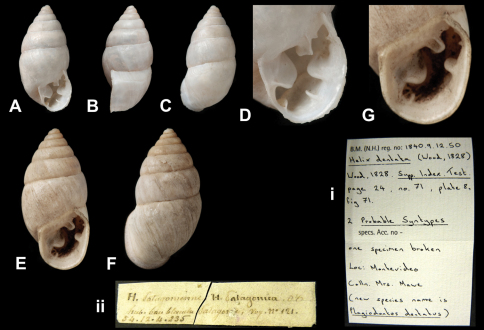
*Plagiodontes* species. **A–D, i** *Plagiodontes dentata* (Wood, 1828), syntype NHM 1840.9.12.50 (H = 17.5) **D** detail of aperture **E–G, ii** *Plagiodontes patagonica* (d’Orbigny, 1835), paralectotype NHM 1854.12.4.235 (H = 22.3) **G** detail of aperture.
